# Two Subgroups within the GH43_36 α-l-Arabinofuranosidase Subfamily Hydrolyze Arabinosyl from Either Mono-or Disubstituted Xylosyl Units in Wheat Arabinoxylan

**DOI:** 10.3390/ijms232213790

**Published:** 2022-11-09

**Authors:** Kai P. Leschonski, Svend G. Kaasgaard, Nikolaj Spodsberg, Kristian B. R. M. Krogh, Mirjam A. Kabel

**Affiliations:** 1 Novozymes A/S, Biologiens Vej 2, 2800 Kongens Lyngby, Denmark; 2 Laboratory of Food Chemistry, Wageningen University, Bornse Weilanden 9, 6708 WG Wageningen, The Netherlands

**Keywords:** phylogeny, substrate specificity, arabinoxylan degradation, synergy, GH51, GH62

## Abstract

Fungal arabinofuranosidases (ABFs) catalyze the hydrolysis of arabinosyl substituents (Ara) and are key in the interplay with other glycosyl hydrolases to saccharify arabinoxylans (AXs). Most characterized ABFs belong to GH51 and GH62 and are known to hydrolyze the linkage of α-(1→2)-Ara and α-(1→3)-Ara in monosubstituted xylosyl residues (Xyl) (ABF-m2,3). Nevertheless, in AX a substantial number of Xyls have two Aras (i.e., disubstituted), which are unaffected by ABFs from GH51 and GH62. To date, only two fungal enzymes have been identified (in GH43_36) that specifically release the α-(1→3)-Ara from disubstituted Xyls (ABF-d3). In our research, phylogenetic analysis of available GH43_36 sequences revealed two major clades (GH43_36a and GH43_36b) with an expected substrate specificity difference. The characterized fungal ABF-d3 enzymes aligned with GH43_36a, including the GH43_36 from *Humicola insolens* (*Hi*ABF43_36a). Hereto, the first fungal GH43_36b (from *Talaromyces pinophilus*) was cloned, purified, and characterized (*Tp*ABF43_36b). Surprisingly, *Tp*ABF43_36b was found to be active as ABF-m2,3, albeit with a relatively low rate compared to other ABFs tested, and showed minor xylanase activity. Novel specificities were also discovered for the *Hi*ABF43_36a, as it also released α-(1→2)-Ara from a disubstitution on the non-reducing end of an arabinoxylooligosaccharide (AXOS), and it was active to a lesser extent as an ABF-m2,3 towards AXOS when the Ara was on the second xylosyl from the non-reducing end. In essence, this work adds new insights into the biorefinery of agricultural residues.

## 1. Introduction

Arabinoxylan (AX) is one of the major plant cell wall polymers present in monocotyledon biomass and can be utilized via enzymatic degradation for the biofuel or prebiotic industries [1,2]. AX composition and content is highly variable among various botanical species and tissue types. For instance, corn bran constitutes up to 50% hemicellulose consisting of mainly AX [3], whereas wheat bran constitutes up to 23 to 32% AX, and wheat endosperm only constitutes up to 2 to 4% AX [4]. Furthermore, different AX populations have varying substitution patterns and, based on water solubility, AX is generally separated into water-extractable and water-unextractable fractions [4,5].

Structurally, AX consists of a β-(1→4)-xylopyranosyl backbone which can be monosubstituted or disubstituted with α-l-arabinofuranosyl (Ara) at *O*-2 and *O*-3 positions [6,7], where α-(1→2) monosubstituted xylosyl is less common in AX from wheat kernels [5]. Besides arabinosyl, ferulic acid, acetyl, and (4-*O*-methyl-) α-d-glucuronic acid substituents are also present to a lesser extent [5,6]. Ferulic acid forms ester bonds with arabinosyl substituents at the *O*-5 position, which can then form covalent diferulic bridges to link arabinoxylan chains together [5]. Acetyl is esterified to the *O*-2 and *O*-3 positions of the xylosyl backbone and (4-*O*-methyl-) α-d-glucuronic acid at its *O*-2 positions [5,6].

The complete enzymatic degradation of arabinoxylan requires the synergistic action of various debranching and xylan-hydrolyzing enzymes, because of its heterogenous substituent composition. In particular, arabinofuranosidases (ABFs) (EC 3.2.1.55) are glycoside hydrolases that catalyze the hydrolytic release of arabinosyl substituents [8]. Fungal ABFs are categorized in the Glycoside Hydrolase (GH) families 43, 51, 54, and 62 based on amino acid sequence similarity in the CAZy database [9]. Fungal GH51, 54, and 62 ABFs mainly release arabinose from monosubstituted xylosyl residues (mXyl) [10], whereas some GH51 ABFs have been found to also act on arabinosyl from disubstituted xylosyl residues (dXyls), but exclusively when substituted on the terminal non-reducing end [8,11].

Nevertheless, only five ABFs that remove arabinose from internal dXyls have been recognized so far, all in GH43. Of these ABFs, four cleave specifically α-(1→3)-Ara from dXyls (ABF-d3) [8,10,12], two are bacterial (GH43_10 active as ABF-d3 from *Bifidobacterium adolescentis* [13] and *Ruminiclostridium josui* [14]), and two are fungal (GH43_36 from *Humicola insolens* [8,12] and *Chrysosporium lucknowense* [15]). Furthermore, recently, one bimodular bacterial GH43 from *Bifidobacterium longum* with an unclassified subfamily was discovered that cleaves α-(1→2)-Ara from dXyls (ABF-d2) [16].

These ABF-d3 and ABF-d2 enzymes act in synergy with other ABFs that act on monosubstituted residues. For example, synergistic action between GH51 from *Meripilus giganteus* and the GH43_36 from *Humicola insolens* on wheat arabinoxylan resulted in 24% more arabinose released [8]. Other fungal GH43 ABFs are found in subfamilies GH43_21, GH43_26, and GH43_29, which are so far characterized to be exclusively active towards mXyls [17,18,19,20,21,22]. A list of currently characterized fungal GH43 ABFs is shown in Table 1.

With only two characterized fungal ABF-d3 enzymes (in GH43_36) so far, we hypothesized that a subgroup of GH43_36 aligns with ABF-d3 activity, and this study sought to employ phylogenetic analysis to identify a new candidate with a different function to this subfamily and to biochemically characterize this candidate. Based on the phylogenetic analysis, a novel GH43_36 candidate from *Talaromyces pinophilus* was selected and heterologously produced and characterized by examination of its product profiles from AX and specific AXOS by ^1^H-NMR, HPAEC-PAD, and MALDI-TOF MS. Furthermore, its catalytic rate and specificity were compared to previously characterized fungal GH51, GH62, and GH43_36 ABFs.

## 2. Results

### 2.1. Structure-Based Phylogenetic Analysis of GH43_36 Reveals Further Subdivision with Distinct GH43_36a and GH43_36b Subgroups

**Figure 1:**
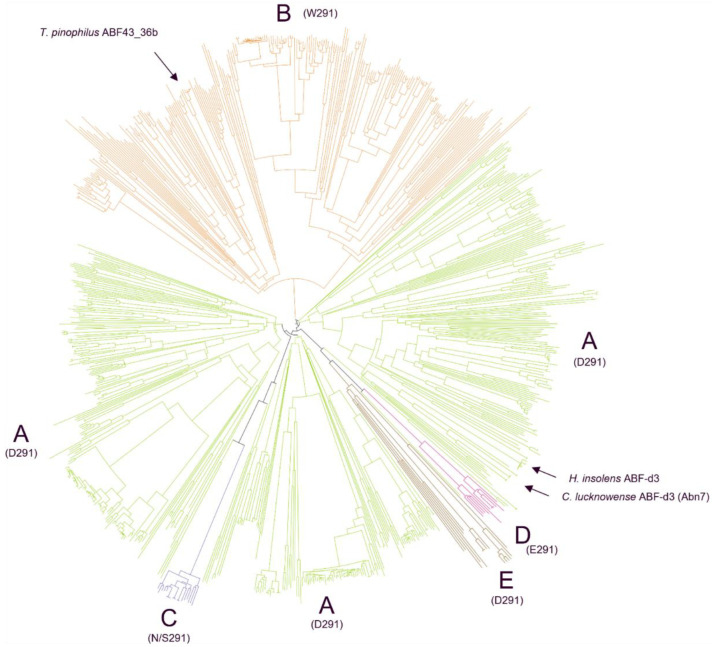
Phylogenetic relations of publicly available GH43_36 sequences of the Ascomycota phylum separated into two main clusters. Cluster 1 contains clades A, C, D, and E, and cluster 2 is defined by clade B. In clade A (green), all candidates have an Asp at position 291, and clade A contains the currently characterized ABF-d3s (*H. insolens* and *C. lucknowense*). Clade B (orange) candidates have Trp at position 291, and the *Tp*ABF43_36b used in this study is part of this clade. Clade C (dark purple) candidates have Asn or Ser at position 291, while clade D candidates (pink) have Glu, and clade E (brown) candidates have Asp at position 291.

**Figure 2:**
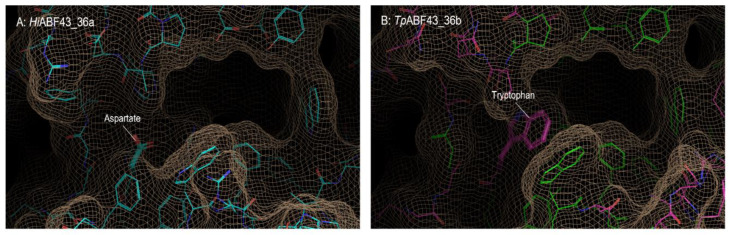
Structures of the active sites of (**A**) *Hi*ABF43_36a and (**B**) *Tp*ABF43_36b homology modeled on the *Hi*ABF43_36a chain (PDB:3ZXJ_B). In panel B, green lines show residues that are identical to amino acids present in *Hi*ABF43_36a shown in panel A, pink lines show residues that are different.

For this study, about 810 publicly available GH43_36 sequences of the phylum Ascomycota were used for the phylogenetic analysis. This phylogeny showed that these GH43_36s were subdivided into two main clusters, cluster 1 contained the major clade A and minor clades C, D, and E, whereas cluster 2 was defined by the other major clade B (Figure 1). Nevertheless, multiple sequence alignment of a subset of representative GH43_36 sequences from clades A and B exhibited high overall similarity between the two clades with general conservation of the residues involved in substrate interaction (SI 1, Figure S1). A noticeable exception was the amino acid positioned at D291 of *H. insolens* ABF-d3 (SI 1, Figure S1). Interestingly, in clade A (in cluster 1), harboring *H. insolens* ABF-d3, for nearly all 500 sequences studied, exclusively aspartate (Asp) was found in position 291 (D291). In contrast, in clade B (in cluster 2) the nearly 250 sequences studied showed a tryptophan (Trp) at the equivalent position (W291) (Figure 1 and Figure 2). In the remaining 7% of the sequences making up several smaller clades, either Asp, glutamic acid (Glu), aspargine (Asn), or serine (Ser) were found at position 291 (Figure 1).

Based on the crystal structure of *Hi*ABF43_36a (PDB:3ZXJ_B), the fungal ABF-d3 contains two pockets within its active site, a catalytic main-pocket and a shallower side-pocket in which D291 is positioned [12] (Figure 2A). Surprisingly, a D291A mutation has previously been found to render the *H. insolens* ABF-d3 fully inactive [12], which points to the importance of D291 for the ABF-d3 function. It was speculated that the Asp/Trp partition between GH43_36 from clades A and B might reflect functional differentiation, hypothesized to result in the loss of activity towards dXyls for the GH43_36b enzymes. Modeled structures of two GH43_36 enzymes from clade A (*Hi*ABF43_36a) and clade B (*Tp*ABF43_36b) used in this study are displayed in Figure 2.

Obviously, a single amino acid position in a protein never solely results in clustering. Indeed, several positions showed a preferential amino acid correlating with either the A or the B clade. Close to the pKa modulator E216 [12], position 218 of the A clade contained either alanine (Ala) or Ser, while in the B clade an Asn was almost exclusively found (SI 1, Figure S1). Although other similarities were found, none of the other positions with a preferential pattern for either the A or the B clade were as strictly conserved as the above-mentioned amino acids at positions 291 and 218. All these other positions appeared to define structural elements that were located away from the active site.

Phylogenetic clustering within an enzyme family of broad taxonomic origin often indicates a functional differentiation, as seen in the Ascomycetes phylogenetic tree (Figure 1). Nevertheless, a phylogenetic tree of about 52 credible public Basidiomycete GH43_36 sequences produced an imperfect clustering, yet still shows a major A clade having Asp at position 291 and a minor B clade with Trp or Ser at an equivalent position (SI 2, Figure S2). Furthermore, the nearly 393 publicly available bacterial GH43_36 sequences all carry an Asp at the 291 position (SI 3, Figure S3), as found for the fungal clade A sequences.

### 2.2. Production, Purification, and General Characteristics of TpABF43_36b

One ABF43_36b from clade B (see Section 2.1) was mainly selected for its availability. It originates from an Ascomycete well known for producing carbohydrases with a significant impact in the hydrolysis of plant cell walls, *Talaromyces pinophilus* [23] (*Tp*ABF43_36b). The gene for this *Tp*ABF43_36b was heterologously expressed in a fungal production host. As a fungal host, an *Aspergillus oryzae* strain was used which had been optimized for monocomponent enzyme production [24]. After induced expression, the cultivation broth was sterile-filtered and subsequently purified by column chromatography. Subsequently, the SDS-PAGE of the *Tp*ABF43_36b (for sequence see SI 1, Figure S1) showed a thick band at 55 kDa (SI 4, Figure S4), which was comparable to the theoretical Mw of 57178.11 Da.

### 2.3. GH43_36a Cleaves α-(1→3)-Ara from Disubstituted Xyls (ABF-d3) and GH43_36b Cleaves Ara from Monosubstituted Xyls

**Figure 3:**
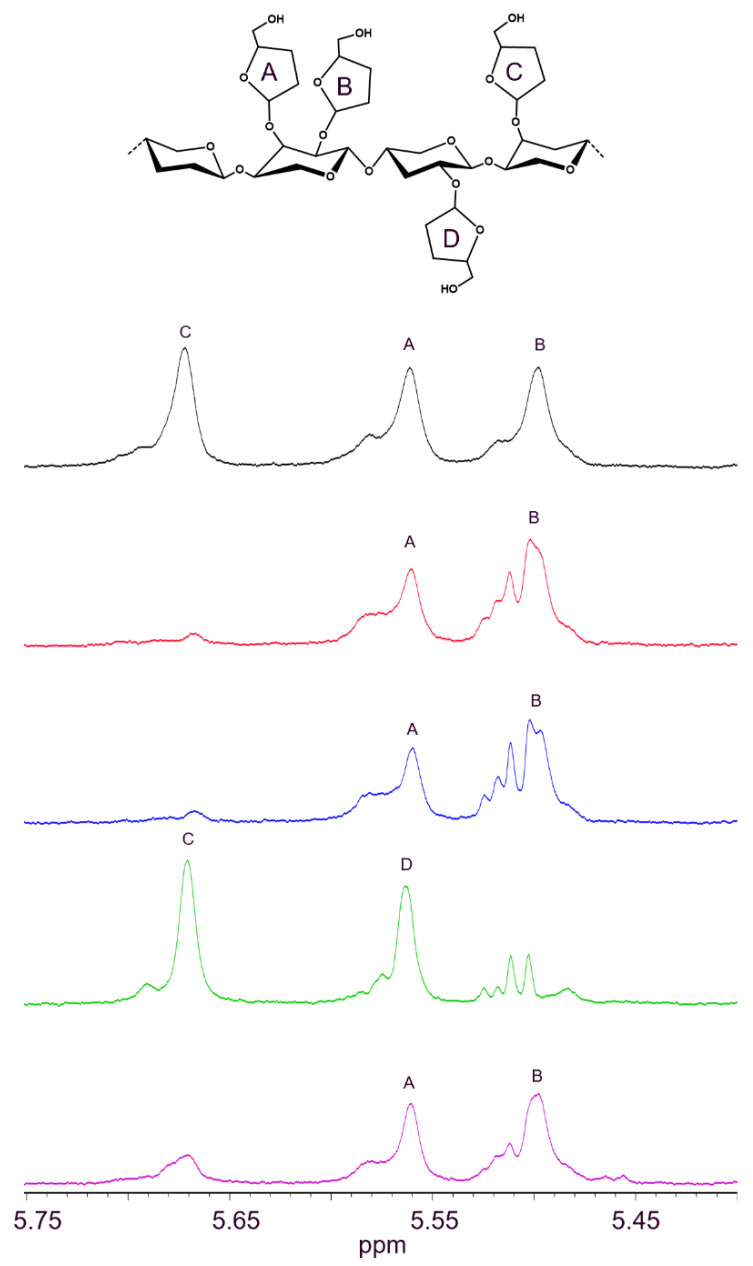
^1^H-NMR spectra of arabinoxylan (AX) incubated without enzyme (black) and with *Mg*ABF51 (red), *Po*ABF62 (blue), *Hi*ABF43_36a (green), and *Tp*ABF43_36b (purple). The 50 μg mL^−1^ enzyme was incubated with 1 mg mL^−1^ AX for 1 h. A: α-(1→3)-Ara from dXyl; B: α-(1→2)-Ara from dXyl; C: α-(1→3)-Ara from mXyl; D: α-(1→2)-Ara from mXyl. Chemical shifts of the ^1^H-protons belonging to the different arabinosyls present in AX were identified by comparison with previous research [7,11]. Reaction conditions are displayed in Section 4.2.2, exp. # 1.

**Figure 4:**
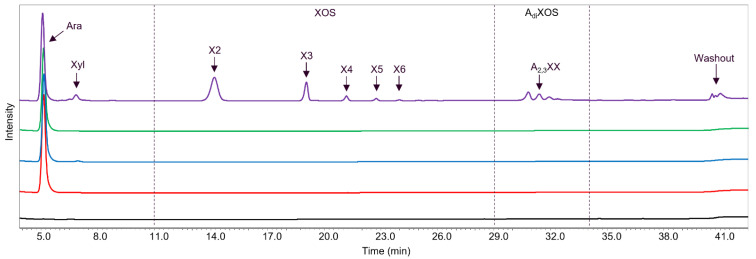
HPAEC reaction product profiles of AX incubated for 24 h without enzyme (black) and with 50 μg mL^−1^ *Mg*ABF51 (red), *Po*ABF62 (blue), *Hi*ABF43_36a (green), and *Tp*ABF43_36b (purple). Arabinose (Ara), xylose (Xyl), XOS (DP 2 to 6), and disubstituted AXOS (A_di_XOS) were detected. Reaction conditions are displayed in Section 4.2.2, exp. # 1.

The activity and specificity towards arabinoxylan (AX) of two ABF43_36s, *Hi*ABF43_36a and *Tp*ABF43_36b, and for comparison of one GH51 and one GH62 ABF (i.e., *Mg*ABF51 and *Po*ABF62, respectively) were analyzed. Thereafter, corresponding AX-digests were subjected to NMR (Figure 3). Chemical shifts of the ^1^H-protons belonging to the different arabinosyls present in AX were identified by comparison with previous research [8,12]. All enzymes tested were active as arabinofuranosidase (ABF) on AX. GH43_36a from *Humicola insolens* hydrolyzed α-(1→3)-Ara from disubstituted xylosyl (dXyl) as expected based on previous research [8,11,12]. This was demonstrated by the loss of both chemical shifts from dXyls and the detection of a new chemical shift representing the α-(1→2)-Ara from monosubstituted xylosyls (mXyls) (Figure 3). Interestingly, the GH43_36b ABF from *Talaromyces pinophilus* hydrolyzed α-(1→3)-Ara from mXyls and showed no activity towards dXyls, similar to the tested GH51 and GH62 enzymes (Figure 3). Regarding arabinosyl distribution in the AX used, slightly more α-(1→3)-Ara was released compared to α-(1→2)-Ara after incubation with *Hi*ABF43_36a (ratio 1:0.87). Thus, AX incubated without enzyme contained about 36% Ara from mXyls and 64% Ara from dXyls. Nevertheless, the dXyl amount was likely slightly overestimated as AX has been reported to contain little amounts of *O*-2 monosubstituted Xyls [25] from which the chemical shift overlaps with α-(1→3)-Ara from dXyl (Figure 3). Still, the presence of *O*-2 monosubstituted Xyl is rare in AX from wheat [5]. Furthermore, small amounts of *O*-2 monosubstituted AXOS were detected after incubation of AX with GH10 and GH11 endoxylanases. Arabinose release was confirmed by HPAEC-PAD (Section 2.4, Figure 4).

### 2.4. Main and Side Activities of TpABF43_36b and HiABF43_36a on AX and AXOS

**Figure 5:**
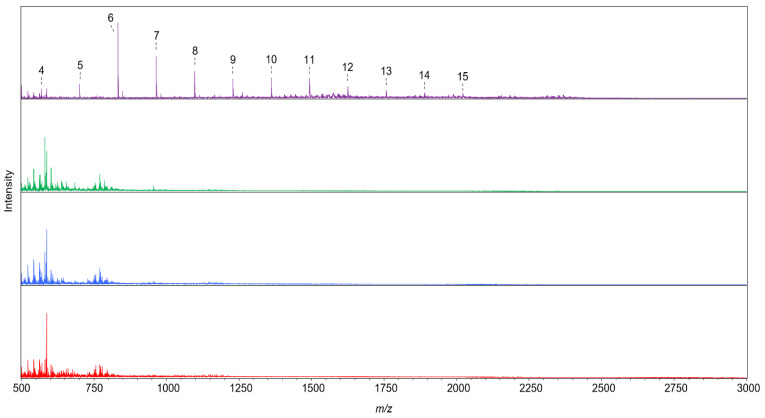
MALDI-TOF mass spectra from AX incubated for 24 h with 50 μg mL^−1^ *Mg*ABF51 (red), *Po*ABF62 (blue), *Hi*ABF43_36a (green), and *Tp*ABF43_36b (purple). All detected Na^+^ adducts of pentose oligomers ((A)XOS; DP 4 to 15) were annotated and numbered 4 to 15. No oligosaccharides were detected for the incubations with *Mg*ABF51, *Po*ABF62, and *Hi*ABF43_36a. Reaction conditions are displayed in Section 4.2.2, exp. # 1.

The reaction product profiles from AX generated by *Tp*ABF43_36b, *Hi*ABF43_36a, *Mg*ABF51, and *Po*ABF62 were analyzed by HPAEC and MALDI-TOF MS (Figure 4 and Figure 5, respectively). *Mg*ABF51, *Po*ABF62, and *Hi*ABF43_36a mainly released arabinose from AX (Figure 4), showing that these enzymes possessed, predominantly, ABF activity. *Po*ABF62 also released xylose, albeit in minor amounts (Figure 4). Likely, it could act on the xylan-chain in an exo-acting matter after arabinosyl substituents were removed, yet only at a low rate. The co-occurrence of these two functionalities is not uncommon and has been previously reported among ABFs, nevertheless not yet among GH62 ABFs [10,26,27]. *Mg*ABF51 seemed to release slightly more arabinose compared to *Po*ABF62 after 24 h incubation (153 and 127 μg mL^−1^, respectively), whereas after 1 h incubation, arabinose release was nearly identical for the two GH51 and GH62 enzymes used (127 and 122 μg mL^−1^, respectively). In comparison, *Hi*ABF43_36a released 117 μg mL^−1^ arabinose after 24 h (Figure 4) from dXyls. As the combination of *Hi*ABF43_36a and *Mg*ABF51 should, in theory, remove all arabinosyl substituents from the AX, a theoretical concentration of 380 μg mL^−1^ arabinose released (~38% total arabinose in AX; supplier information) was expected from their combined activity. This was indeed close to the theoretical combined activity from the sum of arabinose released by *Hi*ABF43_36a and *Mg*ABF51 when acting alone on AX after 24 h incubation (384 μg mL^−1^). Nevertheless, the combined arabinose release of a mixture of *Hi*ABF43_36a and *Mg*ABF51 after 1 h incubation was found to be incomplete, as only ~320 μg mL^−1^ arabinose was released (see Section 2.6.3).

As a further comparison, *Tp*ABF43_36b released 119 μg mL^−1^ arabinose after 24 h, and, thus, released slightly lower amounts of arabinose compared to *Mg*ABF51 and *Po*ABF62 after 24 h incubation (Figure 4).

More surprisingly, the new *Tp*ABF43_36b substantially released xylo-oligosaccharides (XOS) and arabinoxylo-oligosaccharides (AXOS), besides arabinose (Figure 4 and Figure 5), while the other ABFs did not. All enzymes were produced in the same production host, and purified, hence, it was not expected that this oligomer release was due to impurities (i.e., other enzymes co-produced and ending up in the *Tp*ABF43_36b fraction used). To confirm this, proteomic analysis was carried out and no xylanase was identified (SI 5, Table S1). Nevertheless, in comparison to a well-characterized GH10 endoxylanase from *Aspergillus aculeatus* (*Aa*Xyn10), the xylanase-like activity of *Tp*ABF43_36b was only 0.3% compared to *Aa*Xyn10 (based on insoluble AZCL-xylan; results not further shown).

In summary, these findings demonstrated that the novel *Tp*ABF43_36b was mainly active as ABF-m2,3 with minor endoxylanase activity, albeit with a relatively low rate compared to the other ABFs tested and the *Aa*Xyn10 endoxylanase.

### 2.5. HiABF43_36a Cleaves α-(1→2 or 3)-Ara from Xyls Disubstituted on the Non-Reducing End and α-(1→2 and 3)-Ara Monosubstituted on the Second Xyl from the Non-Reducing End

**Figure 6:**
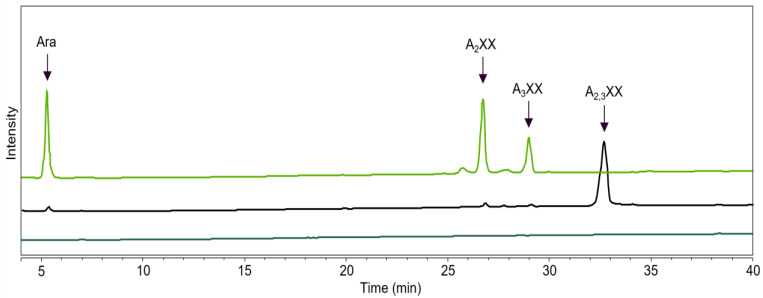
HPAEC profiles of A_2,3_XX (xylotriose disubstituted on the non-reducing end) incubated with *Hi*ABF43_36a (green), without enzyme (substrate blank) (black), and with *Hi*ABF43_36a but without substrate (enzyme blank) (dark green). Incubation contained 1 μg mL^−1^ enzyme and 100 μg mL^−1^ A_2,3_XX. Reaction conditions are displayed in Section 4.2.2, exp. # 2.

The specificities of the GH43_36 ABFs were further studied with selected AXOS. The well-characterized *Hi*ABF43_36a (ABF-d3) revealed a so far unknown specificity in this study, highlighted by the release of both α-(1→2)-Ara and α-(1→3)-Ara from A_2,3_XX with 100% conversion (Figure 6). In this oligomer, the disubstitution is located on the non-reducing terminal end, which might have resulted in the release of either arabinoses.

**Figure 7:**
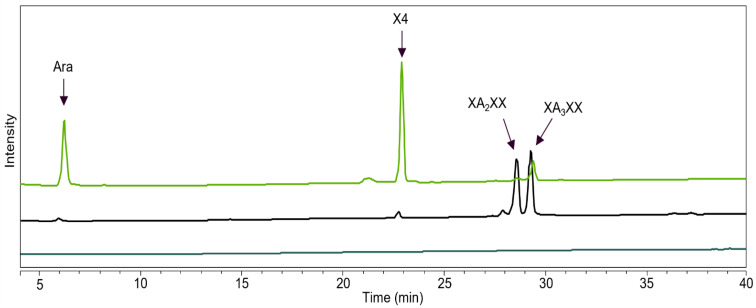
HPAEC profiles of a mixture of XA_2_XX and XA_3_XX (xylotetraose *O*-2 and *O*-3 monosubstituted on the second xylosyl from the non-reducing end, respectively) incubated with *Hi*ABF43_36a (green), without enzyme (substrate blank) (black), and with *Hi*ABF43_36a but without substrate (enzyme blank) (dark green). Incubation contained 25 μg mL^−1^ enzyme and 100 μg mL^−1^ XA_2_XX/XA_3_XX mixture. Reaction conditions are displayed in Section 4.2.2, exp. # 2.

Incubation of *Hi*ABF43_36a with a mixture of XA_2_XX and XA_3_XX revealed another novel specificity, namely the cleavage of arabinose from monosubstituted AXOS (Figure 7). The ABF-m2,3 activity was unexpected and not in line with activity on AX (Section 2.3, Figure 3) and previous results from GH43_36a ABFs [8,12]. Based on our results, we propose that *Hi*ABF43_36a can cleave arabinose from mXyls specifically when monosubstituted on the second xylosyl from the non-reducing end. This ability might be the result of a structural similarity between AXOS monosubstituted on the second Xyl from the non-reducing end and AXOS disubstituted on the non-reducing end, as Xyl and Ara are spatially similar [27,28]. The same specific arabinose release was observed for *Hi*ABF43_36a on an endoxylanase digest from AX (produced by GH11 from *Thermomyces lanuginosus*; *Tl*Xyn11) that was pretreated with an excess of *Hi*ABF43_36a (ABF-d3) to remove disubstitutions. GH11 enzymes are endoxylanases that require three consecutive unsubstituted backbone units for activity and can only cleave the chain between two unsubstituted Xyls [25,29]. Hence, the resulting AXOS mainly have arabinosyl substituents on the second xylosyl from the non-reducing end (i.e., XAXX) [25,29]. Subsequently, the resulting AXOS were subjected to a second incubation with excess *Hi*ABF43_36a. This second incubation even released ~40% arabinose compared to the total arabinose still present in these AXOS, which were basically devoid of dXyls, pointing at the relevance of this, so far, unnoticed side activity. Total arabinose release was achieved from these AXOS by *Mg*ABF51 ABF-m2,3 (SI 6, Figure S5).

### 2.6. Rate and Synergy of the GH43_36, GH51, and GH62 Enzymes used in this Study

#### 2.6.1. Rate of TpABF43_36b, HiABF43_36, MgABF51, and PoABF62 Enzymes on AX

**Figure 8:**
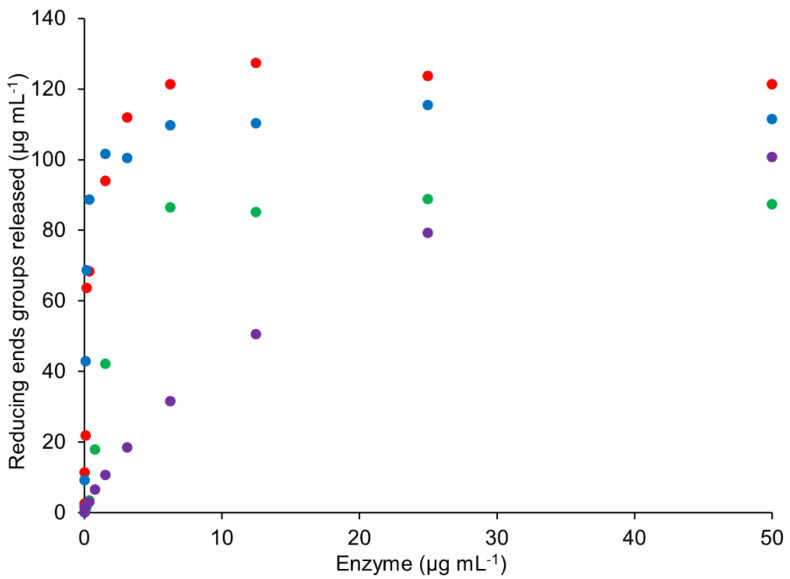
Reducing end groups released from 1 mg mL^-1^ AX by *Mg*ABF51 (red), *Po*ABF62 (blue), *Hi*ABF43_36a (green), and *Tp*ABF43_36b (purple) after 1 h incubation. Reaction conditions are displayed in Section 4.2.2, exp. # 3 and 4.

Commonly, ABF (GH51) rates are determined with *p*-nitrophenyl α-l-arabinofuranoside, but GH62 and GH43_36 ABFs, generally, do not show activity on such artificial substrates [8,10,30]. Therefore, reaction rates of *Tp*ABF43_36b, *Hi*ABF43_36a, *Mg*ABF51, and *Po*ABF62 acting on AX were compared by reducing end groups released with increasing enzyme concentration (Figure 8). *Mg*ABF51 and *Po*ABF62 showed the fastest rates of all ABFs tested, followed by *Hi*ABF43_36a.

The novel *Tp*ABF43_36b showed an even lower reaction rate compared to the *Hi*ABF43_36a and, apparently, did not reach maximum activity after 1 h incubation (Figure 8). In hopes of finding a more suitable substrate for *Tp*ABF43_36b, its activity on arabinan, rhamnogalacturonan, and insoluble corn fiber was investigated, however, no arabinose was released.

#### 2.6.2. Rate of TpABF43_36b, HiABF43_36, MgABF51, and PoABF62 on Specific AXOS

**Figure 9:**
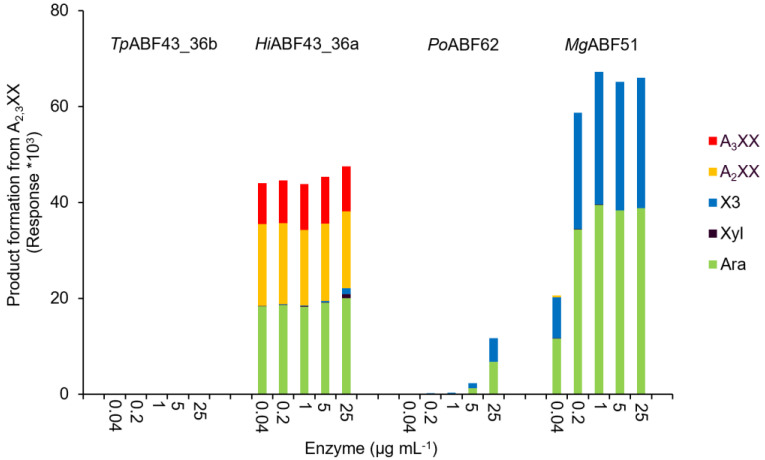
A_2,3_XX product formation (HPAEC) when incubated with *Hi*ABF43_36a, *Tp*ABF43_36b, *Po*ABF62, and *Mg*ABF51. Arabinose (Ara), xylose (Xyl), xylotriose (X3), A_2_XX, and A_3_XX were detected. Reaction conditions are displayed in Section 4.2.2, exp. # 2.

**Figure 10:**
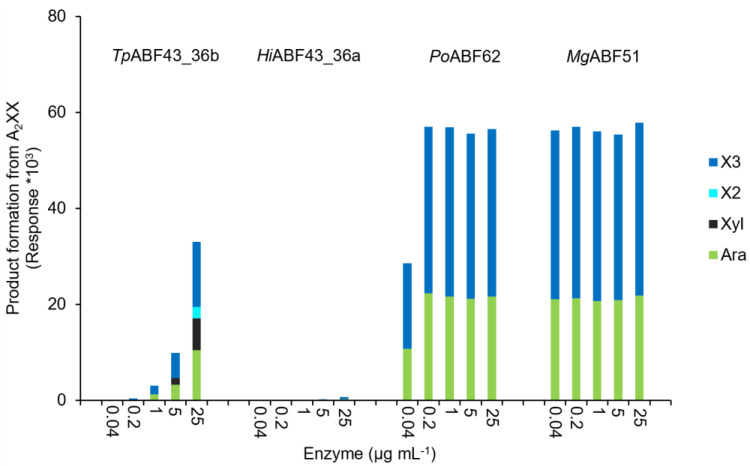
A_2_XX product formation (HPAEC) when incubated with *Hi*ABF43_36a, *Tp*ABF43_36b, *Po*ABF62, and *Mg*ABF51. Arabinose (Ara), xylose (Xyl), xylobiose (X2), and xylotriose (X3) were detected. Reaction conditions are displayed in Section 4.2.2, exp. # 2.

**Figure 11:**
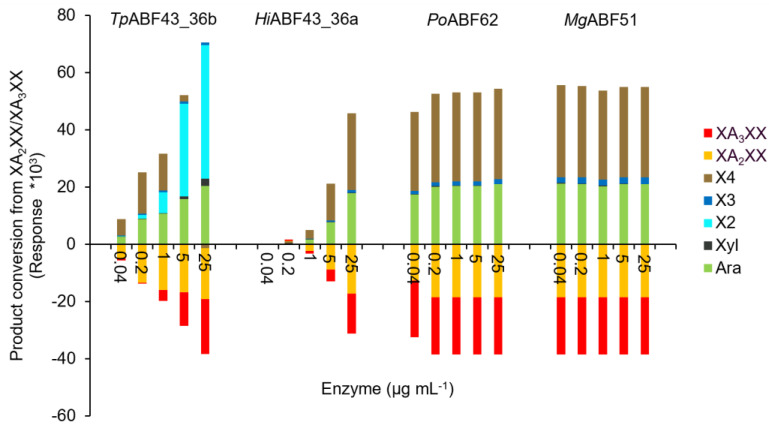
XA_2_XX/XA_3_XX product conversion (HPAEC) when incubated with *Hi*ABF43_36a, *Tp*ABF43_36b, *Po*ABF62, and *Mg*ABF51. Arabinose (Ara), xylose (Xyl), XOS (DP 2 to 4), XA_2_XX, and XA_3_XX were detected. Reaction conditions are displayed in Section 4.2.2, exp. # 2.

The rates and specificities of *Tp*ABF43_36b, *Hi*ABF43_36a, *Po*ABF62, and *Mg*ABF51 enzymes on specific AXOS were examined by incubation with A_2,3_XX, A_2_XX, and a mixture of XA_2_XX and XA_3_XX (Figure 9, Figure 10 and Figure 11, respectively).

The *Po*ABF62 and *Mg*ABF51 were highly active on all monosubstituted AXOS (Figure 10 and Figure 11), which was expected as they act on mXyls from AX and AXOS as their preferred substrate [8,11,31]. Nevertheless, *Mg*ABF51 showed significant activity with the full conversion of A_2,3_XX to arabinose and xylotriose at 1 μg mL^−1^ (Figure 9). The latter is in line with previous research, as for some GH51 ABFs it has been reported that they cleave arabinose from dXyls exclusively when substituted on the non-reducing end [11]. The *Mg*ABF51 required at least 25 times more protein loading to achieve 100% conversion of A_2,3_XX compared to A_2_XX and XA_2_XX/XA_3_XX, indicating that its action towards dXyls on the non-reducing end can be considered as a side activity. In addition, no remaining A_2_XX or A_3_XX were detected from the product profile of the A_2,3_XX digest (Figure 9), as these compounds were immediately hydrolyzed to arabinose and xylotriose due to high ABF-m2,3 preference. Unexpectedly, *Po*ABF62 converted a small amount of disubstituted compound to arabinose and xylotriose (Figure 9), but only at high enzyme concentration.

As shown above (Section 2.5), *Hi*ABF43_36a had mainly ABF-d3 activity, but could also cleave to either α-(1→2)-Ara or α-(1→3)-Ara from AXOS disubstituted on the non-reducing end (Figure 9) and could cleave arabinose when monosubstituted on the second xylosyl from the non-reducing end (Figure 11). Furthermore, *Hi*ABF43_36a showed 100% arabinose release from A_2,3_XX at the lowest enzyme concentration tested (Figure 9). This high reaction rate of *Hi*ABF43_36a towards A_2,3_XX suggested that AXOS disubstituted on the non-reducing end could be its preferred substrate over AX. Calculations were made to compare the reaction rate of *Hi*ABF43_36a on A_2,3_XX and AX. The *Hi*ABF43_36a was almost twice as fast when acting on dXyls from A_2,3_XX compared to AX (SI 7). The calculation was performed by extrapolation of the reaction conditions, and, thus, is a rough estimate. Nevertheless, this calculation suggested that *Hi*ABF43_36a prefers either AXOS in general or specifically AXOS disubstituted on the non-reducing end over AX.

In line with our previous results, *Tp*ABF43_36b was active towards A_2_XX, XA_2_XX, and XA_3_XX, but only with low rates compared to *Mg*ABF51 and *Po*ABF62 enzymes, yet with a higher rate compared to *Hi*ABF43_36a (Figure 10 and Figure 11). In addition, *Tp*ABF43_36b was more active on XA_2_XX compared to XA_3_XX (Figure 11), suggesting that its preferred substrate might contain more *O*-2 substituted Xyls. Nevertheless, after cleaving arabinosyl from the monosubstituted AXOS, the formed xylotetraose could be further hydrolyzed to mainly xylobiose, which confirms the endoxylanase activity already observed towards AX by *Tp*ABF43_36b (Figure 4, Section 2.4). Furthermore, minor xylosidase activity was also observed by the release of minor amounts of xylose from either xylotriose or xylotetraose, which is also in line with previous activity by *Tp*ABF43_36b on AX (Figure 4, Section 2.4). It seems that *Tp*ABF43_36b prefers arabinofuranosidase activity over xylanase activity, or xylanase activity is hindered by substituents since no substituted arabinoxylobiose was formed from XA_2_XX and XA_3_XX (Figure 11). The *Tp*ABF43_36b was not active on A_2,3_XX (Figure 9).

#### 2.6.3. Synergy of TpABF43_36b, HiABF43_36, MgABF51, and PoABF62 on AX

**Figure 12:**
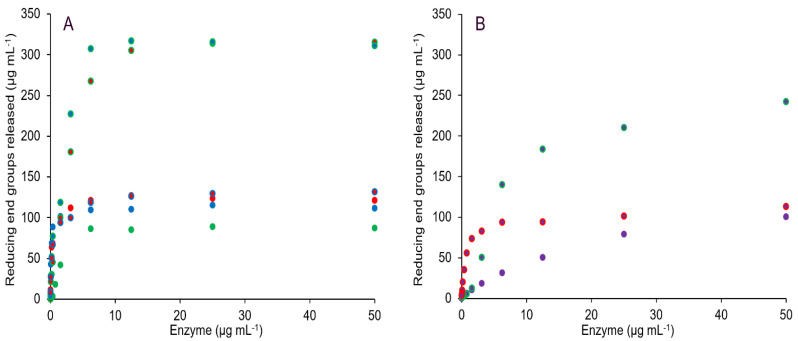
Reducing end groups released from AX (1 mg mL^−1^) shows synergy after 1 h incubation with (**A**) *Hi*ABF43_36a (green), *Mg*ABF51 (red), *Po*ABF62 (blue), and their combined activities, and (**B**) *Tp*ABF43_36b separately (purple) and together with *Mg*ABF51 (red) and *Hi*ABF43_36a (green). Reaction conditions are displayed in Section 4.2.2, exp. # 3 and 4.

The synergy between *Tp*ABF43_36b, *Hi*ABF43_36, *Mg*ABF51, and *Po*ABF62 on AX was analyzed by reducing end groups released from the hydrolysis of AX. The synergy between *Hi*ABF43_36a with *Mg*ABF51 or *Po*ABF62 enzymes was expected as the action of *Hi*ABF43_36a generates more mXyls for *Mg*ABF51 and *Po*ABF62 to act on [8,11,12,31,32,33]. Indeed, ~54% more arabinose was released by their combination (Figure 12A). Synergy experiments were also performed for *Tp*ABF43_36b with *Hi*ABF43_36a and *Mg*ABF51 (Figure 12B). *Tp*ABF43_36b performed as expected based on our previous results on AX, and acted in synergy with *Hi*ABF43_36a, nevertheless with a relatively low reaction rate compared to *Hi*ABF43_36a and *Mg*ABF51. The synergy of *Tp*ABF43_36b with *Hi*ABF43_36a resulted in ~56% more arabinose released (SI 8, Figure S6), whereas no synergy was observed between *Mg*ABF51 and *Tp*ABF43_36b.

## 3. Discussion

Previously, only two fungal GH43_36 arabinofuranosidases (ABFs) have been characterized that are exclusively active towards α-(1→3)-arabinose from disubstituted xylosyl (dXyl), one from *Humicola insolens* (*Hi*ABF43_36a) [8,12] and the other from *Chrysosporium lucknowense* (Abn7) [15]. In the current study, phylogenetic analysis of available sequences within the GH43_36 ABF subfamily of Ascomycetes showed a further subdivision into two main clusters, where cluster 1 consisted of major clade A and minor clades C, D, and E, and cluster 2 which consisted of the second major clade B. A functional activity difference was expected between the two clusters based on the topology of their active site. Most notably, GH43_36 members from clade A (GH43_36a) contain an aspartate (D291) in the side-pocket of their active site, which is essential for the aforementioned specificity towards dXyls [12], whereas GH43_36 members from clade B (GH43_36b) contain a tryptophan at equivalent position (W291). Nevertheless, a less conserved active site topology was found for the two corresponding clades in the Basidiomycetes GH43_36 phylogeny.

In this research, the product profiles from AX and specific AXOS of a novel fungal GH43_36 enzyme that aligns with clade B (*Tp*ABF43_36b) were examined and compared to previously well-characterized GH51 (*Mg*ABF51), GH62 (*Po*ABF62), and clade A GH43_36 (*Hi*ABF43_36a) ABFs. An overview of specificities and reaction rates is shown in Table 2.

The *Hi*ABF43_36a was active towards dXyls as its preferred substrate (Table 2). Activity towards disubstituted xylosyl was expected as GH43_36a enzymes contain two pockets in the active site, in which the secondary pocket interacts with the α-(1→2)-Ara to direct the α-(1→3)-Ara into the catalytic main-pocket [8,11,12]. This is different compared to so far characterized GH51 or GH62 ABFs, which only have a single pocket allowing for the entry of one arabinosyl substituent into the active site, thus hydrolyzing monosubstituted residues [8,31,32,33]. As expected, based on the above-described mechanism, synergy for *Hi*ABF43_36a together with *Mg*ABF51 or *Po*ABF62 resulted in 54% more arabinose released from AX. Accordingly, *Hi*ABF43_36a was highly active on A_2,3_XX and showed no activity on A_2_XX (Table 2). Surprisingly, *Hi*ABF43_36a generated both A_2_XX and A_3_XX from A_2,3_XX (Figure 6), even though GH43_36a ABFs were thought to be exclusively active on α-(1→3)-Ara from disubstituted residues [8,12]. Most likely, selectivity towards α-(1→3)-Ara is lost for Xyl units disubstituted on the non-reducing terminal end. This selectivity is likely exclusive to fungal GH43_36a enzymes, as the bacterial GH43_10 from *Bifidobacterium adolescentis* only generated A_2_XX from A_2,3_XX [11]. Furthermore, the reaction rate of *Hi*ABF43_36a towards A_2,3_XX was almost double compared to the reaction rate on AX, suggesting that *Hi*ABF43_36a might act in synergy with xylanases. Another unexpected selectivity was demonstrated as *Hi*ABF43_36a hydrolyzed monosubstituted arabinose from XA_2_XX and XA_3_XX (Table 2). Based on our results, it was speculated that *Hi*ABF43_36a can hydrolyze AXOS monosubstituted on the second xylosyl from the non-reducing end as they are structurally similar to AXOS disubstituted on the non-reducing terminal end (i.e., when the non-reducing end Xyl is seen as one of the Aras on A_2,3_XX). Nevertheless, reaction rates on XA_2_XX and XA_3_XX were lower compared to A_2,3_XX and AX, indicating that activity towards these monosubstituted AXOS is a side activity. Furthermore, the bacterial GH43_10 from *Bifidobacterium adolescentis* was not able to hydrolyze arabinose from XA_2_XX [11] and the GH43_36a from *Chrysosporium lucknowense* showed no activity on GH10 endoxylanase digest of AX pretreated with ABF-d3 [15]. Therefore, the ability to hydrolyze AXOS monosubstituted on the second xylosyl from the non-reducing end is likely not a common functionality among ABF-d3 enzymes. Surprisingly, the newly discovered specificities of *Hi*ABF43_36a towards A_2,3_XX, XA_2_XX, and XA_3_XX were different compared to the observed specificities of *Hi*ABF43_36a by Sørensen et al. [8], where *Hi*ABF43_36a’s action on AXOS produced by GH10 or GH11 endoxylanases only released α-(1→3)-Ara.

Unlike previously characterized ABF-d3 GH43_36a enzymes [8,12,15], *Tp*ABF43_36b cleaved arabinose exclusively from monosubstituted residues and showed minor endoxylanase side activity (Table 2). Loss of ABF-d3 activity was expected for GH43_36b enzymes compared to GH43_36a, based on the topology of their active site. GH43_36b enzymes are structurally similar to GH43_36a, yet they have a tryptophan, whereas GH43_36a have an aspartate at an equivalent position (D291) which is essential for activity towards dXyls [12]. In fact, synergy was observed between *Tp*ABF43_36b and *Hi*ABF43_36a which resulted in 56% more arabinose released from AX. This, along with ^1^H-NMR results from AX, confirmed that *Tp*ABF43_36b was active as ABF-m2,3. Besides ABF activity, *Tp*ABF43_36b also possessed minor endoxylanase activity as a side activity. Endoxylanase activity was predicted to be a property of GH43_36 enzymes in previous research [12], where mutation of a tyrosine residue on the rim of the active site of *Hi*ABF43_36a opened its pocket and introduced endoxylanase activity while keeping its ability to act on disubstituted residues [12]. This suggested that GH43_36 enzymes already possess the catalytic apparatus for endoxylanase activity, which was confirmed here. Nevertheless, the presence of the tryptophan in the active site does not open the catalytic pocket of *Tp*ABF43_36b. Instead, it is expected that the xylanase activity of *Tp*ABF43_36b could be induced via stacking interactions with the xylan backbone, as *Tp*ABF43_36b contains two tryptophan residues in close proximity (Figure 2, Section 2.1), which is a feature also used by xylan-binding family 15 carbohydrate-binding modules [34]. Moreover, despite the relatively low reaction rate of *Tp*ABF43_36b on AX compared to other ABFs tested in this study, its inactivity on arabinan, rhamnogalacturonan, and insoluble corn fiber suggested that the β-(1→4)-linked xylan backbone with simple arabinosyl substituents (i.e., AX from wheat) is its preferred substrate.

The *Mg*ABF51 and *Po*ABF62 showed the fastest rates out of all ABFs tested and released arabinosyl from both α-(1→2) and α-(1→3) monosubstituted Xyls from all substrates (Table 2). The *Mg*ABF51 also hydrolyzed arabinosyl from the disubstituted A_2,3_XX, yet at a lower rate, indicating that this is a side activity. In literature, some GH51 ABFs have been determined to also hydrolyze disubstituted residues exclusively when substituted on the terminal non-reducing end [11]. Overall, *Mg*ABF51 showed slightly higher rates on AXOS compared to *Po*ABF62, yet they have similar activity on AX (Table 2).

## 4. Materials and Methods

### 4.1. Materials

#### 4.1.1. Chemicals and Substrate

Ammonium acetate (NH_4_Ac), Triton X-100, l-arabinose, xylose, potassium chloride (KCl), 50% sodium hydroxide (NaOH, for HPLC), sodium acetate (NaAc), Sodium chloride (NaCl), 2,4,6-trihydroxyacetophenone (THAP), glucose, maltose, maltodextrins mixture (DP 3–30), potassium sodium tartrate (KNa-tartrate), para-hydroxy benzoic acid hydrazide (PAHBAH), acetonitrile (ACN), D_2_O (99.9 atom% D), and methanol were purchased from Sigma Aldrich (St. Louis, Missouri, USA). MilliQ was prepared with a MILLI-Q ACADEMIC 230V/50HZ purchased from Merck (Darmstadt, Germany). HCl and NaOH were purchased from Merck (Darmstadt, Germany). Low-viscosity wheat arabinoxylan (AX), arabinan (AB) from sugar beet, rhamnogalacturonan (RG) from soybean, azurine-cross-linked (AZCL) xylan (beechwood), 3-α-l-plus 2-α-l-arabinofuranosyl-xylotetraose (XA_3_XX/XA_2_XX) mixture, 2,3-di-α-l-arabinofuranosyl-xylotriose (A_2,3_XX), 3-α-l-arabinofuranosyl-xylotetraose (XA_3_XX), 2-α-l-arabinofuranosyl-xylotriose (A_2_XX), xylobiose (X2), xylotriose (X3), xylotetraose (X4), xylopentaose (X5), and xylohexaose (X6) were purchased from Megazyme (Bray, Wicklow County, Ireland). Insoluble corn fiber, defatted by hexane extraction and destarched by amylase treatment, was supplied by Novozymes A/S (Lyngby, Denmark). Finally, 10 mM NH_4_Ac buffer was prepared and adjusted to pH 5.0 using 2 M HCl.

#### 4.1.2. Enzymes

The following heterologously produced enzymes were supplied and purified by Novozymes A/S (Kongens Lyngby, Denmark): GH43_36a from *Humicola insolens* (*Hi*ABF43_36a, GenBank: CAL81199.1, 10.55 mg mL^−1^), GH43_36b from *Talaromyces pinophilus* (*Tp*ABF43_36b, TREMBL: A0A6V8HEJ6, 3.28 mg mL^−1^), GH51 from *Meripilus giganteus* (*Mg*ABF51, GenBank: CAL81200.1, 4.48 mg mL^−1^), GH62 from *Penicillium oxalicum* (*Po*ABF62, UniProt: A0A2R4KZP3, 9.86 mg mL^−1^), GH10 from *Aspergillus aculeatus* (*Aa*Xyn10, TREMBL: A0A8G1VVX7, 2.62 mg mL^−1^), and GH11 from *Thermomyces lanuginosus* (*Tl*Xyn11, SWISSPROT: O43097, 7.6 mg mL^−1^). Protein concentrations of *Hi*ABF43_36a, *Mg*ABF51, *Po*ABF62, *Aa*Xyn10, and *Tl*Xyn11 were determined by measured absorbance at 280 nm divided by their calculated molar extinction coefficient, whereas the protein concentration of *Tp*ABF43_36b was determined by the bicinchoninic acid (BCA) protein assay kit (Thermo Fisher Scientific, Waltham, MA, USA) performed according to manufacturer’s instructions.

### 4.2. Enzyme Characterization

#### 4.2.1. Phylogenetic Analysis of GH43_36 from Ascomycetes

Fungal Ascomycete GH43_36 sequences were obtained from the publicly available genome and protein sources: EMBL, ENA, NCBI, JGI, and UniProt. Genome sequences for which proteomes were not available were subjected to assembly by SPAdes [35]. Gene calling was performed using GeneMark ES [36] or Augustus [37]. Complete and credible GH43_36 gene models were aligned using Clustal Omega multiple sequence alignment service version 1.2.4 [38]. Visualization of the phylogenetic tree was created with iTOL as a rooted tree without outgroup and leaf sorting [39].

#### 4.2.2. Enzyme Incubations

Table 3 shows the incubation conditions of the different experiments performed by number, denoted as # in the figure captions. Almost all enzyme incubations with arabinoxylan (AX) and arabinoxylo-oligosaccharides (AXOS) were performed in single determination, except the incubation with only *Hi*ABF43_36a in exp #3, which was performed in duplicates. Experiment #1 was performed in 2 mL Eppendorf tubes, whereas all other experiments were performed in 96 well plates (Thermo Fisher Scientific, Waltham, MA, USA). All experiments were performed while shaking (600 rpm) in 10 mM NH_4_Ac buffer pH 5.0 at 40 °C. Incubations varied in enzyme type and concentration, substrate type and concentration, incubation time, and analysis method (Table 3). AXOS (1 mg mL^−1^) and AX, AB, and RG stocks (2 mg mL^−1^) were prepared in 10 mM NH_4_Ac buffer pH 5.0, in which AX and RG were dissolved by heating to ~75 °C. Insoluble corn fiber was added to the incubation as suspension (30 mg mL^−1^) while stirring. All incubations included a blank of pure MilliQ (not incubated), enzyme blanks (only of the highest protein concentration), and substrate blanks. Samples from experiment #1 were heat inactivated at 99 °C for 15 min after incubation before being subjected to lyophilization and subsequent ^1^H-NMR (Section 4.2.3). Samples subjected to HPLC (Section 4.2.4) and MALDI-TOF MS (Section 4.2.5) from experiment #1 were filtered in 0.5 mL 10 kDa centrifugal filter units (Merck, Darmstadt, Germany) by centrifugation (14,500× *g* rpm, 20 min), whereas incubations from experiment #2 to 6 were filtered in 10 kDa 96 well filter plates (Pall corporation, New York, USA) by centrifugation (1500× *g*, 45 min). Analysis with PAHBAH from experiments #1 to 4 followed immediately after incubation and were not inactivated or filtered, whereas samples from experiments #5 and 6 were filtered in 10 kDa 96 well filter plates (Pall corporation, New York, NY, USA) by centrifugation (1500× *g*, 45 min). Samples were stored and frozen before analysis if not analyzed immediately.

#### 4.2.3. Analysis of ABF Activity with ^1^H-NMR

Samples (~1.5 mL) were lyophilized overnight and redissolved in 600 μL D_2_O, and inserted in NMR-tubes (Duran, Duisburg, Germany). ^1^H NMR spectra were recorded at 60 °C with a 400 MHz Bruker Avance III^TM^ HD spectrometer equipped with a BBFO room temperature probe. ^1^H-NMR spectra were recorded at 400 MHz using 128 scans. Data analysis was performed by means of TopSpin 3.6.

#### 4.2.4. Analysis of Reaction Products with HPAEC-PAD

Enzyme activity was analyzed with high-performance anion exchange chromatography (HPAEC). An ICS3000 HPLC system (Dionex, Sunnyvale, CA, USA) was equipped with a pump, an autosampler set to 12 °C, and a pulsed amperometric detector (PAD) equipped with a gold working electrode and an Ag/AgCl reference electrode. The data were processed by means of Chromeleon 7. Samples (10 μL) were loaded onto a CarboPac PA1 Analytical column (4 i.d. mm × 250 mm, 10 μm, Dionex, Sunnyvale, CA, USA) with a CarboPac PA1 Guard pre-column (4 i.d. mm x 50 mm, 10 μm, Dionex, Sunnyvale, CA, USA). The column temperature was set at 30 °C. Three different elution profiles were used, using four types of eluents: MilliQ water (A), 500 mM NaOH (B), 100 mM NaOH + 500 mM NaAc (C1), and 500 mM NaOH + 1.67 M NaAc (C2). Experiments 1, 2, and 6 (Table 3) and samples from Section 4.2.8 were subjected to elution profile 1 (mono- and oligo-saccharides), in which a flow rate of 1000 μL min^−1^ was used, as follows: starting with 12% B, 8 min isocratic at 12% B, 22 min linear gradient to 7.4% B + 23% C1, 8 min linear gradient to 6% B + 30% C1, 1 min linear gradient to 100% C1, 2 min isocratic at 100% C1, 1 min linear gradient to 12% B + 0% C1, 10 min isocratic at 12% B. Samples from experiment no. 3 (Table 3) were subjected to gradient 2 (mono-sugars), in which a flow rate of 800 μL min^−1^ was used, as follows: starting with 3% B, 4.5 min isocratic at 3% B, 2.5 min linear gradient to 7% B, 3 min linear gradient to 14% B + 5% C1, 15 min linear gradient to 8% B + 35% C1, 3.1 min linear gradient to 15% B + 0% C1, 0.9 min linear gradient to 10% B, 1.1 min linear gradient to 3% B, 9.9 min isocratic gradient at 3% B. The samples from experiment no. 4 (Table 3) were subjected to gradient 3 (oligo-saccharides), in which a flow rate of 1250 μL min^−1^ was used, as follows: starting with 20% B, 10 min linear gradient to 18.2% B + 9% C2, 25 min linear gradient to 15% B + 23% C2, 0.5 min linear gradient to 0% B + 100% C2, 2.5 min isocratic at 100% C2, 0.5 min linear gradient to 20% B + 0% C2, 7.5 min isocratic gradient at 20% B.

#### 4.2.5. Analysis of Reaction Products with MALDI-TOF MS

Reaction products were analyzed for their *m/z* values with matrix-assisted laser desorption/ionization time-of-flight mass spectrometry (MALDI-TOF MS). A solution of 20 mg mL^−1^ THAP in 90:10 methanol:MilliQ (*v/v*) was used as a matrix. The 0.7 μL of 1:1 (*v/v*) mix of the sample (Table 3) and matrix were spotted on an AnchorChip^TM^ target plate (Bruker Daltonics GmbH, Bremen, Germany) in duplicates and air-dried. The MALDI-TOF-MS spectra were recorded with an Ultraflextreme MALDI-TOF/TOF machine (Bruker Daltonics GmbH, Bremen, Germany) using FlexControl version 3.4 (Bruker Daltonics GmbH, Bremen, Germany) equipped with a smartbeam Nd:YAG laser operated in positive mode. After a delayed extraction time of 90 ns, ions were accelerated to the kinetic energy of 25 kV and operated in reflector mode with 26.5 kV. The lowest laser power required to obtain good spectra was used (85%). Calibration was performed with glucose, maltose, and a mixture of maltodextrins up to DP 30. A mass scan range of *m*/*z* 100–3000 was used. Data was collected as an average of 6000 shots and processed by means of FlexAnalysis version 3.4 (Bruker Daltonics GmbH, Bremen, Germany).

#### 4.2.6. Analysis of Reducing End Groups with PAHBAH Assay

Reducing end groups were determined using *para*-hydroxy benzoic acid hydrazide (PAHBAH). The PAHBAH reagent was prepared with 15 mg mL^−1^ PAHBAH in 50 mg mL^−1^ KNa-tartrate, and 20 mg mL^−1^ NaOH buffer. The sample was added to the PAHBAH reagent (100:75, *v/v*) and then incubated in sealed PCR-plates (Thermo Fisher Scientific, Waltham, MA, USA) at 95 °C for 10 min followed by cooling to 10 °C (temperature gradient of 3 °C s^−1^) in a T100^TM^ Thermal Cycler from Bio-Rad (Hercules, CA, USA). Absorbance was measured at 405 nm using a SpectraMax Plus 384 microplate reader (GE Healthcare, Chicago, IL, USA) with 100 μL incubated sample in 96 well plates (Thermo Fisher Scientific, Waltham, MA, USA). Quantification was performed with an arabinose calibration curve (0–200 μg mL^−1^).

#### 4.2.7. AZCL-Xylanase Activity

The xylanase potential of *Tp*ABF43_36b was evaluated using insoluble AZCL xylan. AZCL-xylan (beechwood) was suspended in buffer 50 mM NH_4_Ac, 50 mM KCl, 0.01% Triton X-100 pH 5.0 (pH adjusted with 2M HCl) while stirring (0.2% *w*/*v*). Enzyme incubations were performed with 90% (*v*/*v*) substrate suspension and 10% (*v*/*v*) *Tp*ABF43_36b (diluted 1, 5, and 10×) in single determination in 96 well plates (Thermo Fisher Scientific, Waltham, Massachusetts, USA) while shaking (600 rpm) for 1 h at 40 °C with a total volume of 250 μL. *Aa*Xyn10 was used for reference with 1 to 1024× dilutions. After incubation, the plate was centrifuged at 2000× *g* rpm for 2 min. Subsequently, 100 μL supernatant was added to a 96 well plate (Thermo Fisher Scientific, Waltham, MA, USA) and measured at 595 OD (any solubilized AZCL-xylan fragments give absorbance at this wavelength) using a SpectraMax Plus 384 microplate reader (GE Healthcare, Chicago, IL, USA).

#### 4.2.8. Production of AXOS Monosubstituted on the Second Xylosyl from the Non-Reducing End

AXOS monosubstituted on the second xylosyl from the non-reducing end were prepared in three steps. All incubations were performed in 10 mM NH_4_Ac buffer pH 5.0. First, AX (1 mg mL^−1^) was incubated with excess *Hi*ABF43_36a (110.5 μg mL^−1^) for 22 h at 40 °C to remove disubstituted arabinosyl substituents. This was followed by enzyme inactivation at 99 °C for 15 min. Secondly, the monosubstituted AX was dialyzed in 3.5 MWCO dialysis discs (Thermo Fisher Scientific, Waltham, MA, USA) against MilliQ water for 24 h and 2 h while stirring to remove arabinose. Thereafter, two 75 μL samples were taken before and after dialysis as a control and analyzed with acid hydrolysis followed by HPLC. Furthermore, a 100 μL sample was taken after dialysis and incubated with excess *Hi*ABF43_36a (100 μg mL^−1^) for 24 h at 40 °C as control. Lastly, the dialyzed monosubstituted AX was incubated with excess *Tl*Xyn11 (50 μg mL^−1^) for 22 h at 40 °C followed by enzyme inactivation at 99 °C for 15 min. Subsequently, the generated mix of monosubstituted AXOS was incubated with *Hi*ABF43_36a (0 to 50 μg mL^−1^) and *Mg*ABF51 (0 to 10 μg mL^−1^) in single determination in 96 well plates (Thermo Fisher Scientific, Waltham, MA, USA) while shaking (600 rpm) with a total volume of 250 μL at 40 °C for 24 h. Incubated samples were analyzed with PAHBAH (Section 4.2.6) and HPAEC-PAD (Section 4.2.4).

#### 4.2.9. Proteomics: Protein ID

Tryptic digests were prepared by a filter-aided sample preparation (FASP) method. Following digestion, the extracted peptides were analyzed on a nano LC-MS/MS system: Evosep One (Evosep)/timsTOF Pro (Bruker Daltonics GmbH, Bremen, Germany). For protein identification, the data were searched against available internal and public databases using the Mascot search engine (Matrix science, Boston, MA, USA) using Genedata Expressionist software with a 1% False Discovery Rate cutoff. Relative protein concentrations were calculated by label-free quantification from peptide volumes using a Hi3 standard method in Genedata Expressionist.

## 5. Conclusions

Extensive phylogenetic analysis of the GH43_36 subfamily within the Ascomycota phylum revealed the presence of two main clusters. Cluster 1 comprised of the major clade A (GH43_36a) containing the, so far, only two characterized GH43_36 arabinofuranosidases (ABFs) and three minor clades. These two GH43_36a ABFs both cleave α-(1→3)-arabinosyl from disubstituted xylosyl residues (Xyl) (ABF-d3). Cluster 2 was defined by major clade B (GH43_36b), and, at present, only contains uncharacterized sequences. Amino acid sequence alignment from a subset of GH43_36 sequences showed a highly conserved aspartate/tryptophan partition between GH43_36a (D291) and GH43_36b (W291) at amino acid position 291, where D291 is essential for the ABF-d3 activity of the previously characterized clade A *Hi*GH43_36 from *H. insolens* (*Hi*ABF43_36a). Therefore, the D/W291 partition was hypothesized to result in a functional activity difference, most notably in the loss of ABF-d3 activity for GH43_36b enzymes. A novel GH43_36b from *Talaromyces pinophilus* that clustered in clade B (*Tp*ABF43_36b) was heterologously produced and examined for its reaction products on AX and specific AXOS. Indeed, the previously characterized *Hi*ABF43_36a was active as ABF-d3, whereas the novel *Tp*ABF43_36b was exclusively active towards monosubstituted Xyls (ABF-m2,3), and, surprisingly, also showed minor endoxylanase side activity. Moreover, new activities were identified for the *Hi*ABF43_36a, as this ABF was also able to cleave α-(1→2 or 3)-Ara from AXOS disubstituted on the non-reducing end and showed ABF-m2,3 activity exclusively towards AXOS substituted on the second xylosyl from the non-reducing end.

## Figures and Tables

**Table 1:** List of characterized fungal GH43 ABFs described in literature and GH51 and GH62 used in this study plus their reported ABF activity.

Class	Enzyme	Organism	Selectivity	Source
GH43_36	*Hi*ABF43_36a ^1^	*Humicola insolens*	ABF-d3	[8]
	Abn7	*Chrysosporium lucknowense*		[15]
GH43_21	Abf43B	*Penicillium chrysogenum*	ABF-m2,3	[17]
GH43_26	*Cs*Ara	*Chaetomium* sp.	ABF-m2,3	[18]
	Abn4	*Chrysosporium lucknowense*		[19]
	*Hi*Abf43	*Humicola insolens*		[20]
	*Pc*ABF43A	*Penicillium chrysogenum*		[21]
GH43_29	*Tp*Ara43_29	*Talaromyces purpureogenus*	ABF-m2,3	[22]
GH51	*Mg*ABF51 ^1^	*Meripilus giganteus*	ABF-m2,3 ABF-d3 ^2^	[8,11]
GH62	*Po*ABF62 ^1^	*Penicillium oxalicum*	ABF-m2,3	-

^1^ Enzyme was used in this study. ^2^ ABF-d3 activity exclusively for terminal non-reducing end xylosyl.

**Table 2:** Comparison of substrate selectivity and rate of arabinofuranosidase (ABF) and xylanase activity on AX and AXOS by *Hi*ABF43_36a, *Tp*ABF43_36b, *Mg*ABF51, and *Po*ABF62. +++ Excellent rate (used as reference), ++ High rate, + Intermediate rate, +/− Low rate,—trace activity, -- no activity.

Enzyme	ABF		Xylanase		AXOS			
	Selectivity	Rate (AX)	Selectivity	Rate (AX)	A_2,3_XX	A_2_XX	XA_2_XX	XA_3_XX
*Hi*ABF43_36a	ABF-d3	++	--	--	+++	--	+/−	+/−
*Tp*ABF43_36b	ABF-m2,3	+	Endo	+/−	--	+/−	+	+/−
*Mg*ABF51	ABF-2,3 ABF-d3 *	+++	--	--	+	+++	+++	+++
*Po*ABF62	ABF-m2,3	+++	Exo	-	-	++	+++	+++

* ABF-d3 activity exclusively when substituted on the terminal non-reducing end.

**Table 3:** Enzyme incubations with experiment number performed with corresponding incubation time (h), protein concentration (μg mL^−1^), substrate type, substrate concentration (μg ml^−1^), and subsequent analysis method.

No.	Enzyme Name	Time (h)	Enzyme (μg mL^−1^)	Substrate Type	Substrate (μg mL^−1^)	Analysis Method
1	*Mg*ABF51	1, 24	1, 50	AX	1000	^1^H-NMR, HPLC, MS
	*Po*ABF62					
	*Hi*ABF43_36a					
	*Tp*ABF43_36b					
2	*Mg*ABF51	24	0 to 25	A_2_XX, XA_3_XX, A_2,3_XX, XA_2_XX/XA_3_XX	100	HPLC
	*Po*ABF62					
	*Hi*ABF43_36a					
	*Tp*ABF43_36b					
3	*Hi*ABF43_36a	1	0 to 50	AX	1000	PAHBAH, HPLC
	*Mg*ABF51					
	*Po*ABF62					
	*Hi*ABF43_36a + *Mg*ABF51					
	*Hi*ABF43_36a + *Po*ABF62					
	*Mg*ABF51, *Po*ABF62					
4	*Tp*ABF43_36b	1, 23	0 to 50	AX	1000	PAHBAH, HPLC
	*Tp*ABF43_36b + *Hi*ABF43_36a					
	*Tp*ABF43_36b + *Mg*ABF51					
5	*Tp*ABF43_36b	*1*	0 to 25	AB, RG	1000	PAHBAH
6	*Tp*ABF43_36b	*1*	0 to 25	Corn fiber	30.000	PAHBAH, HPLC

## Data Availability

Data is available on request from the corresponding author.

## Supplementary Materials

The following supporting information can be downloaded at: https://www.mdpi.com/article/10.3390/ijms232213790/s1.
