# Supplementary material for: Two Subgroups within the GH43_36 α-l-Arabinofuranosidase Subfamily Hydrolyze Arabinosyl from Either Mono-or Disubstituted Xylosyl Units in Wheat Arabinoxylan
Source: Int J Mol Sci. 2022 Nov 9;23(22):13790. doi: 10.3390/ijms232213790 (PMC9693073; doi:10.3390/ijms232213790)
Supplement: Supplementary file 1 [file ijms-23-13790-s001.zip › ijms-1974645-supplementary.pdf]

Supplementary materials

SI 1. Multiple sequence alignment of a subset of GH43_36a and GH43_36b enzymes

```
                                                                             D43
                                                                             *
A__A_pullulans            ---MkfssVfaALtavgcaLA----------qqTyTNPVlWyDlADiDVFRkGn--qYYY
A__H_insolens             ---MlglkVLclsAvvgtaVSVphagnlPrqASTFTNPVlWEDhpDleVFRvGs--vfYY
A__C_lucknowense          MgrLndliaLlALlsgsatsAavrntasqaRAAeFnNPVlWEDYpDlDVFRvGs--TfYY
A__C_graminicola          -mkLqlssVLlAVvatvva-----------qATFsNPVlWEDlADieViRvnD--TYYY
A__A_oryzae               ---MkvnpfLlgplmsmatrt----------vSqiTNPtfWEDFADlDiiRvdD--TfYY
A__Pseudogymnoascus_sp    --MklaikVffdLAmavmpVl----------gATFTNPVlWEDlADlDiFRvdD--TYYY
A__C_fioriniae            --MrswlrsLvtIAalaapIA----------AeTFTNPVIWEDlADNeVhRvGD--TYYl
A__F_oxysporum            --MrailgLLfSVAaam--------------AekFTNPVIWEDlsDveVtRaGD--aYfm
B__T_pinophilus           -------mLLlgaAvlafgLiak--------AqnFnNPVIygDFpDNDiFlgpDGkTfYf
B__Pseudogymnoascus_sp    MrsisvvvafsSLAsavsnIAVrad------tgTFTNPVVyEDFADNDVsKgpDG-lfYf
B__M_phaseolina           ---MkpaiVLaAIlpiswaa-----------gATFsNPVVyEDFADNDVsvgpDG-lfYf
B__F_langsethiae          ---MkvstILagaSlwaasLtpasaglvP-RAdTFnNPIIysDFpDNDVFlgpDn-nYYf
B__S_alcalophilus         ---MerllLLaSallaaapLSVkascdd---nATFsNPlIysDFpDNDVFlgpDG-aYYf

                              F60                                                W108
                              ^                                                  ^
A__A_pullulans            SASTMsfSPGAPILRSgDLVsWEfIGHSVPrLDFkDtaYDL-adGkqAYvRGIWASsmqY
A__H_insolens             SsSTfaYSPGAPvLkSyDLVhWtpVtHSVPrLNFGsn-YDLPsGtpgAYvKGIWASTLRY
A__C_lucknowense          SsSTfaYSPGAPvLkSyDLVNWtpVtHSVPtLNFGDr-YnLtgGtpagYvKGIWASTLRY
A__C_graminicola          tASTMHYSPGAPvLkSynLVdWEfVGnSVPeLvFGDe-YyL-nGtsrAYvdGIWASsLqY
A__A_oryzae               SASnMHYSPGAPlLkSyDLlNWEfaGHSiPeLtFGse-YyL-eGGnhAYiRGtWASTLnY
A__Pseudogymnoascus_sp    SASnMHYSPGAPILsSqDLVNWQyIGHSVPtLaFGsk-YDm-sGGatsYvKGtyASTmRY
A__C_fioriniae            tASTMHYSPGAPILRSkDLVNWEyVsHSVPvLDwnsq-YDL-adGknAYvKGIWASsLRh
A__F_oxysporum            tASTfHYSPGAPvLRSyDLVNWEhIGHSVPvLDwssk-YsL-enGqrAYvKGIWASsLRY
B__T_pinophilus           SsSTfHYSPGAPILeStDLVNWQvVGHSVPtLDFGgd-Ysm-qnGqtAYnRGIWASTLRh
B__Pseudogymnoascus_sp    SASnMHfSPGAPILRSaDLVNWEmIGHSVPkLaFGDn-YDL--nGgvAYrgGtWASTmRY
B__M_phaseolina           SASnMHYSPGAPILRSyDLaNWElIGHSVPsLDFGEr-YnL--tGgqAYrgGtWASTmRY
B__F_langsethiae          SASnfHYSPGAPILRSkDLlNWDiIGHSiPrLtFGDg-YDL-kGdqrsYrgGtWASsmRY
B__S_alcalophilus         SASnfHYSPGAPILRSdDLVNWDlIGHSlPrLtFGDg-YDLPpGGghsYrgGtWASTLRY

                                                                                     D167
                                                                                    ^*
A__A_pullulans            REydntfYWIGCVdFk--tTYiYkAsdPaG------------tWsKvgeIg--TCYYDCG
A__H_insolens             RrSNdrfYWyGCVE-G--rTYlwTsPggnalanNGEVpPSaWnWqhtAtID--nCYYDaG
A__C_lucknowense          RpSNdkfYWyGCVEFG--kTYiwTssgtragdrDGEVdPAdWvWephppID--rCYYDsG
A__C_graminicola          RESNGlfYWyGCiqsGemkTwVfTAsdPaG------------PWTaqppIN--eCfYDvG
A__A_oryzae               RpStstfYWmGCid-G--tTYiYTAPnadG------------PWTQaStIN--kCYYDCG
A__Pseudogymnoascus_sp    RaSNktWYWyGCiEyG--gTYVYsAPSatG------------PWTQkAkIs--TCYYDaG
A__C_fioriniae            RaSNGlWYfVaCVEFs--kTYVYTsPdPaG------------PWTKagtID--TCYYDvG
A__F_oxysporum            RESdGkfYfVaCiEFG--kTYVYsAsSPtG------------PWsQittIN--kCYYDaG
B__T_pinophilus           RQSNnkWYWIGCidFw--tTYVYTAPdvsG------------PWqKsSsfq--pCfYDCG
B__Pseudogymnoascus_sp    RESdktWYWIGCVnFy--hsfVYTAsSaaG------------PWTQsAeIp--TCYYDCG
B__M_phaseolina           RESNGlWYWIGCVdFw--yTYiYTskSPtG------------PWTRsAqlpgGTCYYDCG
B__F_langsethiae          RKSNGlWYWIGCVnFy--qTwVfTAsSPeG------------PWTnrgnlgDGnChYDnG
B__S_alcalophilus         RESNGlWYWIGCVnFy--hTwVfTAPSPeG------------PWsnnghlgDGnCYYDnG

                                                                                  E216
                                                                                  *
A__A_pullulans            mLfDgqn-iYVAYGNtkISVAQLnnD-ftQks-TtqVySssi----tIEGshMkKiNnrY
A__H_insolens             LLIDDDDTMYIAYGNptINVAQLSpDGtrQVRvqQrVyAhPqgq--tVEGaRMYKirGnY
A__C_lucknowense          LLIDDDDkMYIAYGNpkIeVAeLSdDGLtEVs-SrvVyTpPagt--tIEGsRMYKvgdaY
A__C_graminicola          iLVDDDDTMYaAYGryNITVAQLSpDGLkpVR-kevVwedsvr---ylEGaRfYhiNssY
A__A_oryzae               LLItDDDnMYVAYGNsgISVAeLSdDGLSEVs-SQmVFptddag--ylEGsRfYqhNGnf
A__Pseudogymnoascus_sp    LLIDDDDTMYIAYGNtqlSVAkLStDGLSQVq-TQvVyNtPssig-vlEGsRMYKiNGaY
A__C_fioriniae            LLfDDDDTpYVAYGNtmISVAQLSaDGLtEtR-retVFqtPsdig-alEGaRMYKrdGnY
A__F_oxysporum            LhfDtDnTpYVAYGrgDlhiAQLSkDmktEVK-eQvVlSpPstl--tlEGsRlYKkdnnw
B__T_pinophilus           LLIDDDDTMYVAYGsnNVSVAQLSaDGLtvlK-TQqVFSyPpect-gIEGNRMYKiNGlY
B__Pseudogymnoascus_sp    LhIDnDDTMYVvhGNtNVNmAQLSaDGLSEVR-TeqlFhyPteaq-gIEGNRMYKrNGiY
B__M_phaseolina           LLVDDDDTMYVvYGatDVkVAQLaaDGLSEVK-SQgVFSasdvgqDgIEGNRMYKrNGnY
B__F_langsethiae          iLIDDDDTMYIvsGsgDVrVAQLSkDGfSQVK-SQnVFSnkdigkedIEGNRMYKiNGlY
B__S_alcalophilus         LLIDDDDTMYVvYGseDVhVAQLSqDGfSEVR-SQrVFgrtdvgaDglEGNRMYKiNGlY

                                                                              H272
                                                                              ^
A__A_pullulans            YIfvTQPAsnEY-VLKSnSG-PlGPYEfKiftkapa-SgIqGsGnPHQGSvVDTPNG-DW
A__H_insolens             YILvTRPADaEY-VLrSTtGsPfGPYEartLVsrIq-gPlanaGfaHQGgiVDaPdG-tW
A__C_lucknowense          YILvTRPADaEW-VLrSTSG-PfGPYEqreLVsrIn-gPlsnaGfaHQGgmVDTPdGrsW
A__C_graminicola          YIwlTRPADGQY-VLKadS--PWGPYtehtvLDrMa-SPIPhsGtPHQGgiVDTPNG-DW
A__A_oryzae               YIfvvhPAneEH-VLmSTSG-vfGPYErrlLIsdag-NPVsGaGyPHQGgiVDTPNG-DW
A__Pseudogymnoascus_sp    YIfsTRPAnGQY-VLrSTSG-PfGPYtiKqLLlNLg-SPIPnGGvPHQGgLiqTqgG-aW
A__C_fioriniae            YIflTRPpDGQF-VLKSsS--PWGPYEvKvLaDrIpLSsVPnaGsPHQGSLVDTqNG-DW
```

```
A__F_oxysporum              YIflTRPAtGQY-iaKSsnG-PfGtYDlriiaDNLkLAaaPrsGaPHQGgLiDTPNG-nW
B__T_pinophilus             YILddcPsqGiteiwKSsS--PWGPYtrKiLsNNtp-SPIPGsGsedQGSLiqTPNG-qW
B__Pseudogymnoascus_sp      YILnddggtqstfiyKSTS--PWGPwtyKrLqaNtp-gPlsGGGtPHQGSLieaspd-nW
B__M_phaseolina             YILndhPgDntY-iwKSsS--PWGPYasKiLVqNIq-SPVsGGGaPHQGSLVkTadd-DW
B__F_langsethiae            YvLndsPsnGQtwiwKSkS--PWGPYEkKlLaDrVt-pPInGGnsPHQGSLikTPkG-DW
B__S_alcalophilus           YILndRPggttY-iwKSaS--PWGPYEpKvLVqdVt-pPlaGGnsPHQGSLieTthG-DW

                                  291 D/W
                                  x
A__A_pullulans              YYlSFiDAYPGGRsPaLAPfvfdSqGwPsLk--atnGfaia-nPyPvtsv--pvkSttG-
A__H_insolens               hYvaFmDAYPGGRIPVvAPlrWtaDGwPevVTDsqGrWGtS-YPiPvrgaknateglast
A__C_lucknowense            YYvaFmDAYPGGRIPVvAPlrWtdDGwPevVTDaqGGWGaS-YPvPvetgktvpddgwe-
A__C_graminicola            wYMaFlDAYPsGRIPVLAPlTWdeeGwPhIVTDeaGGWGkt-YPvPvetd--kkvnaaGe
A__A_oryzae                 YYMaFvDAYPGGRIPVLAPlvWGSDGwPsvel-vdGGWstt-YdAvnitppstpiSsfep
A__Pseudogymnoascus_sp      YYMaFvDsYPGGRVPVLAPISWGSDGFPvLqT-vNnaWGsS-YtAPlsl---sptShtG-
A__C_fioriniae              YYMgFTDiYPGGRsPVLAPITWGdDGFPsLVT-vNGaWGdyEYPlPra----dvpSalr-
A__F_oxysporum              YYMSFvDmYPGGRaPaLAPITWGSDGFPkItl-vNGqWGdyDYPlPkr----tvpSpiG-
B__T_pinophilus             YfMSFawAYPsGRLPILAPITWGSDGFPiLqT-vNGaWGaS-YPyPlpqnt-stlSwns-
B__Pseudogymnoascus_sp      YfMSFTwdYPnGRIPVLAPITWGdDdFPiLtT-vdGkWGat-YPAPlptv--etpSwtG-
B__M_phaseolina             YYMSFTwAYPaGRMPVLgPVTWGSDdFPvfVdgsNGGWGvS-YPlPlpah--plpSwtG-
B__F_langsethiae            YfMSFTwAYPaGRLPVLAPVTWGSDGFPiLtkgsNGGWGsS-YPt-lpgtsgvtknwar-
B__S_alcalophilus           YYMSFTwAYPaGRLPVLAPIeWGnDGyPvfVTgdNGGWGtS-YlvPhpemlaspkdwtr-


A__A_pullulans              -TDtFssgkLgPQWEWNHNPDTSKFSfA--agGGLvLKTAsVakDLYhAkNTLTHRIlGP
A__H_insolens               dlDeFrGTrfSehWEWNHNPDTSKFTllGGNeGGLiLRTATVTgDLfaARNTLTrRIaGP
A__C_lucknowense            -lDeFrGgrLShhWEWNHNPDpaRFAlAGGdeGGLvLQaATVTEDLfaARNTLTrRIrGP
A__C_graminicola            YTDtFdGpaLSPQWafNHNPDnSawel---gaeGLTLhTATVTDDLYsARNTLaHRIlGP
A__A_oryzae                 YTDqFtGdtLSPQWEWNHNPDndKwSI---dh-GvTLtTAsVTDDLYaAkNTLTHRIlGP
A__Pseudogymnoascus_sp      -TDaFpGSSLNPQWEWNHNPDTtKFSV---NN-GLiLsTATVTNDLYhARNTLsHRIlGP
A__C_fioriniae              -TDeFaGTSLrPEWEWNHNPDiSgFeV---Nd-GiTLRTvTlTsDLYsARNTLTHRIrGP
A__F_oxysporum              -TDtFpGpnLradWEWNHNPDTksFTV---NN-GLTLKTvsVTkDLYqARNTLTHRIrGP
B__T_pinophilus             -wDgFwGnSLpPtWEWNHNPDltKvSf---NNpGLTLQTATVTNDLYhAqNTLTHRtnGq
B__Pseudogymnoascus_sp      -TDsFnGTSLgPQWEWNHNPDTtKYSV---dN-GvTLsaATVTDDLYkARNTLTHRVhGe
B__M_phaseolina             -TDaFrGTSLgPaWEWNHNPDpSKYAV---NN-GLTLsaATVTDDLYaARNTLTHRIhGe
B__F_langsethiae            -TDtFsGTSLSPsWEWNHNPDTnsFTV---NN-GLTLRTsTVTkDiYqARNTLTHRthGn
B__S_alcalophilus           -TDfFdGTSLdPsWEWNHNPDdnsYvV---dd-GLTLyTAsVTNDLflARNTLTHRthGe


A__A_pullulans              dstaTIqLDiSqMtstDqAGLslFRDnSAYIGIrng----vVILqrditMgaa--W--nT
A__H_insolens               kasGifrLDvrgMrDGDRAGavlFRDraAYIGVwkqGnearIVMvddLrLnEDg-W--rT
A__C_lucknowense            kssGTfrLDvSrMrDGDRAGavlFRDtaAYIGVwkqGDeaTIVVvdGLeLalss-W--tT
A__C_graminicola            kssGTfrLnlgNMADGDvAGLsiFRDeSAYIGfrkaGDSlvlVavhdviasEstgW--qT
A__A_oryzae                 istaTIhLntSsMfsGDRAGLsllRhySAwIGVvnDGgTshIgVttGLeMDsD--W--nT
A__Pseudogymnoascus_sp      tssGTViLnFgNMADGDRAGLAmlRDsSAwIGIrkDGsSikIsMwsGLaMtst--W--AT
A__C_fioriniae              igtGTVhLnvaNMADGDRvGLAAlRDkSswIGIeREGDvYSVVtvgGLtMntD--W--tT
A__F_oxysporum              qgtGTVliDFSkMADGDRtGLAvlRDsSAwIGIeREGsnFnlVfntGLsMntD--W--tT
B__T_pinophilus             fPVGTVqiDFSNMADGDtAGLAlFkelSAwIGVvRsGnTYTItcvqGLtqDpNnhW--AT
B__Pseudogymnoascus_sp      qPVaTIvLDYSNMADGDRcGLAAFRDwtAYIGVvRsGDTYSVVMqeGLtqnstd-W--ST
B__M_phaseolina             fPVGTVaiDFSNlADGDRAGLAAFRDrSAsIGVhRDGDTYTIqVvhGmtqDEst-W--AT
B__F_langsethiae            rPVGTVkinFSNMrDGDRAGfsAFRDqSAYIGIhRtngqFTlatkhGmnMDE---WNGAT
B__S_alcalophilus           fPVGTVeiDFtNlADGDlAGLAAFRDqtAYIaVrRNGneYTlVtrhnmiiDE---WDGSs


A__A_pullulans              lSTGrLetsAs--------lpAkt--tNVWLRLhADIKP----AGThqgvFSYStD-GkS
A__H_insolens               aSTGrVaAngP--------VidtnaqQDiWLRIdADItPAFGtntertTFyYSiDGGrt
A__C_lucknowense            vSTGrVaeTgP--------tlSst--QDVWLRIeADItPAFGtntArttTFSYSvDGGkt
A__C_graminicola            TSnGTVVATAtasdidlsgVadGt--aDVyLRIvADLhPtFGvAaNnpAqllYSiD-Gen
A__A_oryzae                 vSTGTesArAe--------fsgse----iWLRVeADItP---yAGSGq--FSYSlD-Gvt
A__Pseudogymnoascus_sp      SSTGSeVAsAa--------IsgsR----VWLRIyADIhv----gsgkqAnFyYStD-GqS
A__C_fioriniae              nSTGaVVerrP--------lgdaR---DVyLRMvADIRP----gGpGnAvFSYSvD-GqS
A__F_oxysporum              kSTGTVsArqn--------nvSyR---kVyLRVsADIRP----gaAGsAvFSYStD-GnS
B__T_pinophilus             TSTGTVVgTAP--------IsAkQ----VyLqatmDaRa----nGSkqATFkYSlD-Gnt
B__Pseudogymnoascus_sp      vSTGTtVeTAe--------VakGR----iWLRssmDsRg----dGSklvTFqYStD-GtS
B__M_phaseolina             TSkGTtVATAP--------VpgGa--KkVWLRaalDaRa----SGTkaAnFSYSfD-Gde
B__F_langsethiae            TSmGeVkATAa--------VpSGR--ttVWLRIqmDtdP----AGTGntvFSYSwD-Gsk
B__S_alcalophilus           vnpGyeVATAP--------VpSGt--KkVWLRaelDVRP----tGSrdAnFlYSwD-Gqn

                                         W526
                                         ^
A__A_pullulans              FkQLGtpYtmnttyyFFIGYRFGIFNFAeqgLGGSVvVKSFdlalgak------------
A__H_insolens               yTrLGpAfamtNSWryFtGYRFGvFNFsTKSLGGeVkVKgFkmnmi--------------
A__C_lucknowense            FvrLGpAfsmsNtWqyFtGYRFGvFNFATKeLGGeVkVKSFqmqpl--------------
A__C_graminicola            FTQLGpdYwchNrWqyFLafRFavFNyATKALGGSVlVKeFTlqlve-------------
A__A_oryzae                 FTnLGdtYeLnNNWeFFMGYRFGIFNFATtgtGGSVvlnSFvls----------------
A__Pseudogymnoascus_sp      FTQLG-SlvLgsSWeFFpGYRyaIFNFATKALGGSVqisSFTvdapgltvsgstggnppt
A__C_fioriniae              yetvGapfeLyNNWeFFMGYRyGIFNyATKALGGSVrVlSFTna----------------
```

```
A__F_oxysporum              FTnfGstvsLgNDWqFFpGhRyGIlNyATKSLGGSVlVsrFdnk----------------
B__T_pinophilus             FTQLGgAftvsNNWSyFMGYRyaIFNFATKALGGSikVlSFvswd---------------
B__Pseudogymnoascus_sp      FvdLGdAYtmntDWAiFMGYRwGIFNhATtALGGSVllESFTqt----------------
B__M_phaseolina             FeQLGrpYtmwtNWAyFMGYRFGIFNyATKkLGGSVaVsSFSsa----------------
B__F_langsethiae            yetLGpnfkLyNgWAFFIaYRFGIFNyAenSLGGSinVESFSaa----------------
B__S_alcalophilus           FSQLGstYeLydgWAFFIaYRFGIFNyATKSLGGSVkVESFTaa----------------
```

```
A__A_pullulans              ------------------------------------------------------------
A__H_insolens               ------------------------------------------------------------
A__C_lucknowense            ------------------------------------------------------------
A__C_graminicola            ------------------------------------------------------------
A__A_oryzae                 ------------------------------------------------------------
A__Pseudogymnoascus_sp      etistggnpptqttstigtttspatggggtvaqygqcggigytggtacaspytckysndw
A__C_fioriniae              ------------------------------------------------------------
A__F_oxysporum              ------------------------------------------------------------
B__T_pinophilus             ------------------------------------------------------------
B__Pseudogymnoascus_sp      ------------------------------------------------------------
B__M_phaseolina             ------------------------------------------------------------
B__F_langsethiae            ------------------------------------------------------------
B__S_alcalophilus           ------------------------------------------------------------
```

Figure S1. Alignment of a subset of (A) GH43_36a from *Aureobasidium pullulans* (TREMBL:A0A4S9IKX3), *Humicola insolens* (GenBank: CAL81199.1), *Chrysosporium lucknowense* (TREMBL:F2X2F8), *Colletotrichum graminicola* (TREMBL:E3QNL1), *Aspergillus oryzae* (TREMBL:Q2U7D1), *Pseudogymnoascus* sp. (TREMBL:A0A094IEN0), *Colletotrichum fioriniae* (TREMBL:A0A010QED7) and *Fusarium oxysporum* (TREMBL:A0A420TQQ6) and (B) GH43_36b from *Talaromyces pinophilus* (TREMBL:A0A6V8HEJ6), *Pseudogymnoascus* sp. (TREMBL:A0A094FCF4), *Macrophomina phaseolina* (TREMBL:K2RHU9), *Fusarium langsethiae* (TREMBL:A0A0M9ERQ2) and *Sodiomyces alcalophilus* (Acral2 ID 1061698). (*) Active site residue, (^) residue involved in substrate interaction, (x) position 291 (D/W).

SI 2. Phylogenetic relations of GH43_36 Basidiomycetes

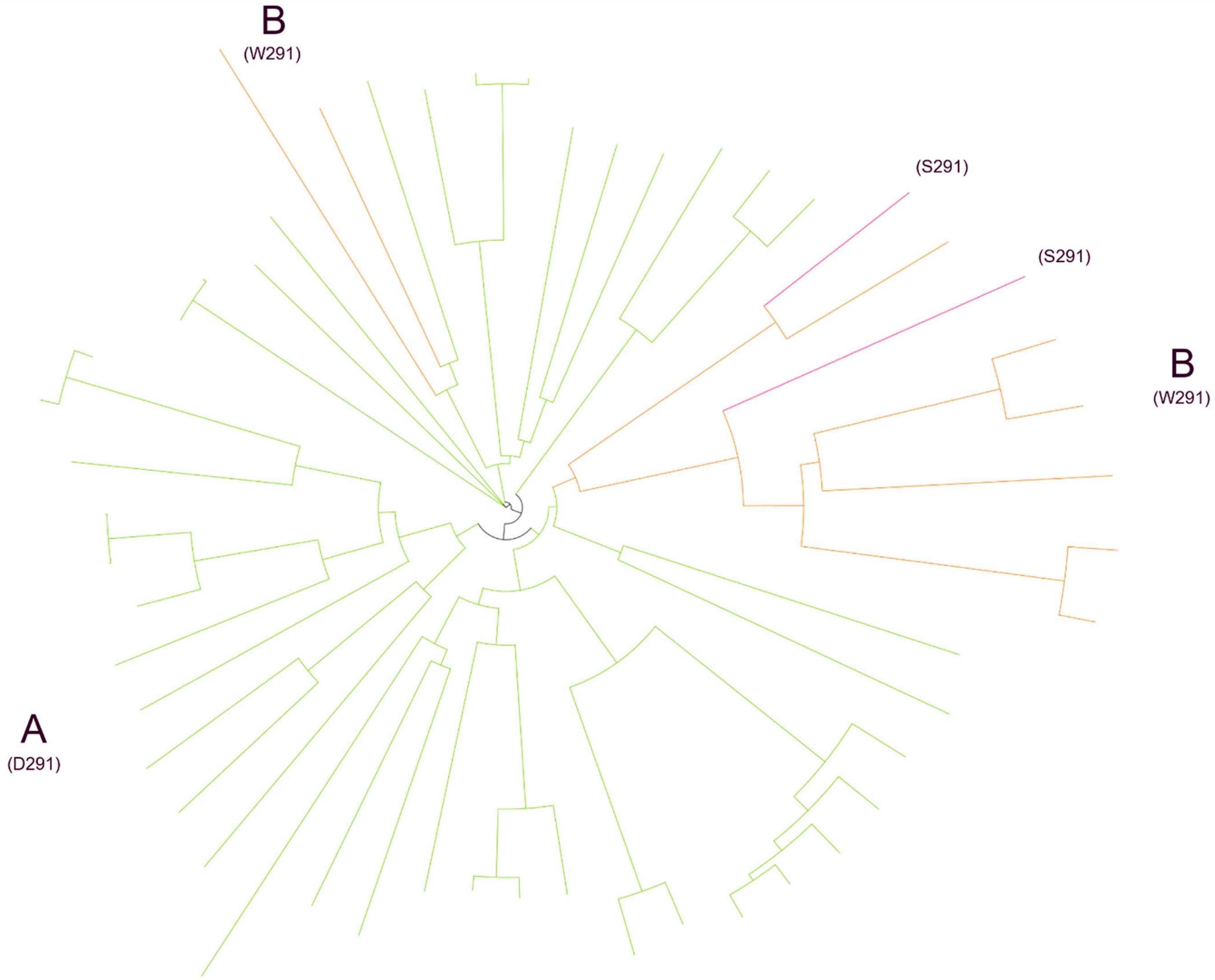

Figure S2. Phylogenetic relations of 52 credible publicly available GH43_36 sequences of the Basidiomycota phylum show imperfect clustering of the two A and B clades found in the GH43_36 Ascomycetes phylogenetic tree. In clade A candidates have an Asp (green) at position 291. In clade B candidates contain a Trp (orange) or Ser (pink) at position 291.

SI 3. Phylogenetic relations of bacterial GH43_36

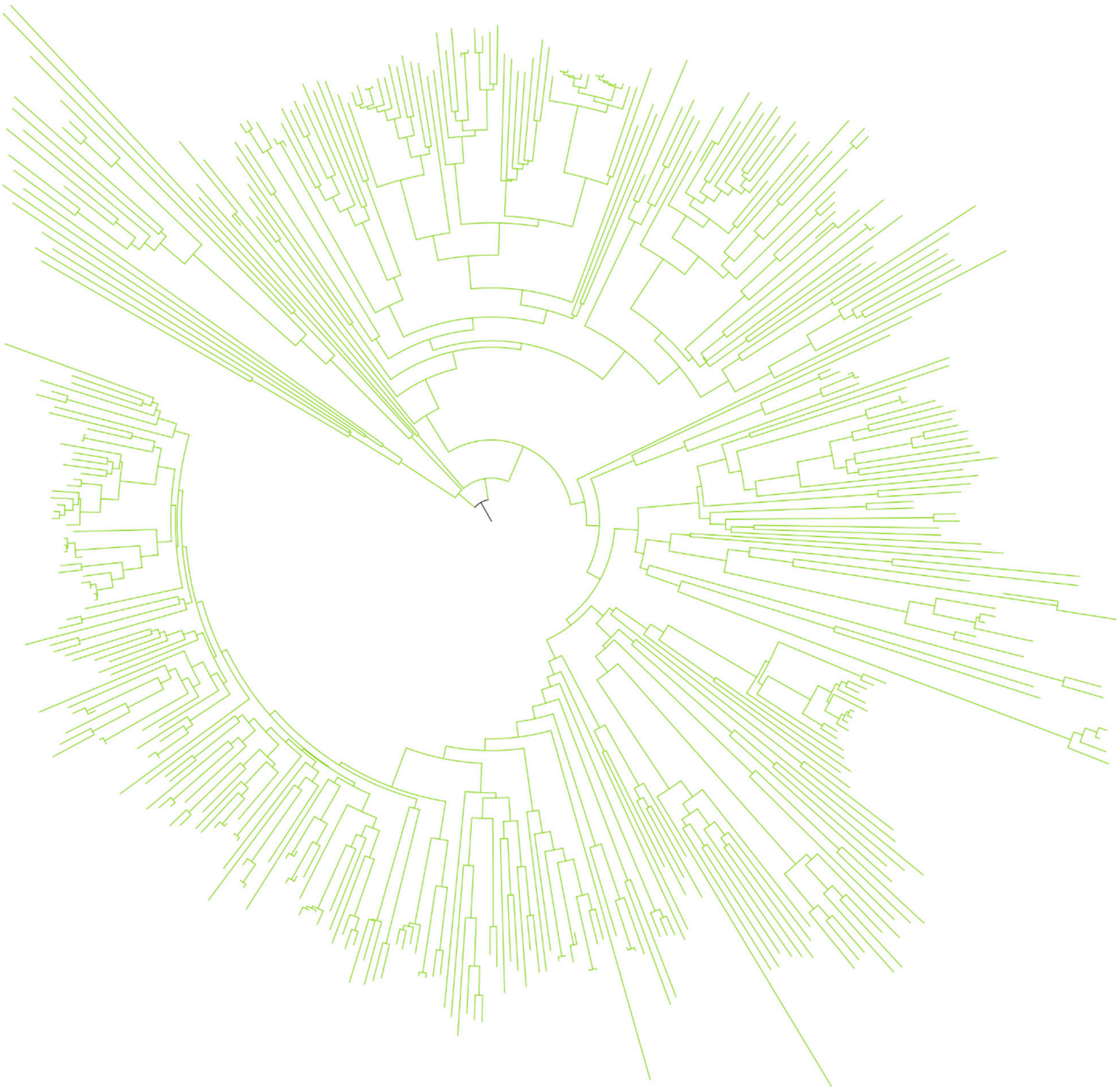

Figure S3. Phylogenetic relations of 393 publicly available GH43_36 sequences from bacteria all cluster in clade A (green) and have an Asp at position 291.

SI 4. SDS-PAGE of TpABF43_36b

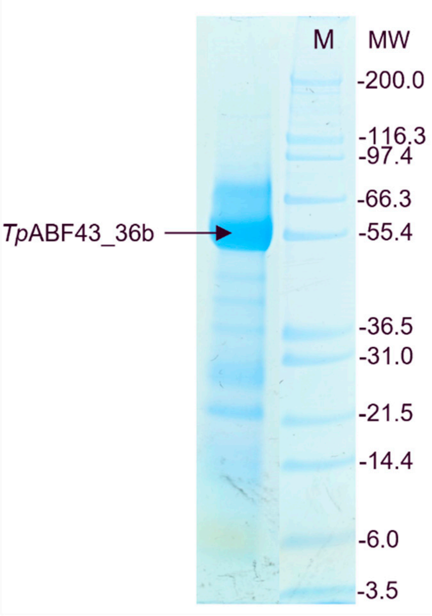

Figure S4. SDS-PAGE of *Tp*ABF43_36b (diluted to 1 mg mL^{-1}) and marker (M) with corresponding MW (kDa).

SI 5. Proteomic analysis of TpABF43_36b

Table S1. Proteomic analysis (protein ID) of *Tp*ABF43_36b. No xylanase was detected.

Name	Description	%
TREMBL:Q2URB2	Uncharacterized protein	*40.78*
NZPROT:P2461D	Di-substituted xylan alpha-1,3-L-arabinofuranosidase GH43_36b	*21.46*
SWISSPROT:P36914	Glucoamylase	*8.47*
TREMBL:Q2U3L3	Uncharacterized protein	*4.26*
TREMBL:Q2UC76	Uncharacterized protein	*4.08*
TREMBL:Q2TYJ8	Uncharacterized protein	*3.64*
TREMBL:Q2U7Y9	Uncharacterized protein	*3.15*
TREMBL:Q2U0Z5	Peptidase S53 domain-containing protein	*2.50*
TREMBL:Q2U068	Uncharacterized protein	*1.94*
TREMBL:Q2UE97	Thioredoxin	*1.59*
TREMBL:Q2UCM3	PABS domain-containing protein	*1.32*
TREMBL:Q2TYE3	Cytochrome b5 heme-binding domain-containing protein	*0.71*
SWISSPROT:Q2UUZ1	Probable glucan endo-1,3-beta-glucosidase eglC	*0.67*
TREMBL:Q2U0P3	Amidase domain-containing protein	*0.67*
TREMBL:Q2UA38	Nucleoside diphosphate kinase	*0.65*
TREMBL:Q2UJW6	D-xylose reductase	*0.56*
TREMBL:Q2UDN5	Uncharacterized protein	*0.49*
TREMBL:Q2U2W9	Uncharacterized protein	*0.35*
TREMBL:Q2U6J1	Peptidyl-prolyl cis-trans isomerase	*0.32*
TREMBL:Q2U426	Transaldolase	*0.29*
TREMBL:Q2UHY1	Aldo_ket_red domain-containing protein	*0.25*
TREMBL:Q2UNJ7	Glycine cleavage system H protein	*0.23*
TREMBL:Q2UIH2	Uncharacterized protein	*0.20*
TREMBL:Q2UM22	Phosphoenolpyruvate carboxykinase (ATP)	*0.16*

TREMBL:Q2U8A3	Uncharacterized protein	*0.16*
TREMBL:Q2TYR8	Peptidylprolyl isomerase	*0.12*
TREMBL:Q2U9Y1	HATPase_c domain-containing protein	*0.11*
TREMBL:Q2TZX5	Aspartate aminotransferase	*0.11*
TREMBL:Q2UJW4	Peptidyl-prolyl cis-trans isomerase	*0.08*
TREMBL:Q2U9C1	Uncharacterized protein	*0.08*
TREMBL:Q2UUQ5	Isocitrate lyase	*0.08*
SWISSPROT:Q9HGY8	Triosephosphate isomerase	*0.07*
SWISSPROT:Q877B6	Superoxide dismutase [Mn], mitochondrial	*0.06*
TREMBL:Q2TZQ3	Uncharacterized protein	*0.06*
TREMBL:Q2U4J7	Semialdhyde_dh domain-containing protein	*0.04*
TREMBL:Q2UHJ0	Dipeptidase	*0.04*
TREMBL:Q2U954	ATP synthase subunit d, mitochondrial	*0.04*
TREMBL:Q2U6S7	Thioredoxin domain-containing protein	*0.03*
SWISSPROT:Q9HGZ2	Glucose-6-phosphate isomerase	*0.03*
TREMBL:Q2USG3	Malate dehydrogenase	*0.03*
TREMBL:Q2TWA0	Peptidase A1 domain-containing protein	*0.03*
TREMBL:Q2UHL9	Citrulline--aspartate ligase	*0.02*
SWISSPROT:Q2ULB2	Mannosyl-oligosaccharide alpha-1,2-mannosidase 1B	*0.02*
TREMBL:Q2UN68	Uncharacterized protein	*0.01*
TREMBL:Q2UM29	Aminotran_1_2 domain-containing protein	*0.01*
TREMBL:Q2U2K8	Adenosylhomocysteinase	*0.01*
TREMBL:Q2UKF3	Fumarate reductase	*0.01*
SWISSPROT:Q2U5P1	Endo-chitosanase C	*0.01*
TREMBL:Q2U5A0	M20_dimer domain-containing protein	*0.01*
TREMBL:Q2TZL2	Uncharacterized protein	*0.01*
TREMBL:Q2U8D2	Lysophospholipase	*0.00*
TREMBL:Q2TWP0	ATP synthase subunit beta	*0.00*
TREMBL:Q2UGW9	FAD-binding PCMH-type domain-containing protein	*0.00*
TREMBL:Q2UJK8	Aspartate transaminase	*0.00*
TREMBL:Q2U008	Uncharacterized protein	*0.00*
SWISSPROT:Q9HGY7	Glyceraldehyde-3-phosphate dehydrogenase	*0.00*
SWISSPROT:Q00248	Protein disulfide-isomerase	*0.00*

SI 6. HiABF43_36a activity towards GH11 digest of arabinoxylan that was pretreated with ABF-d3

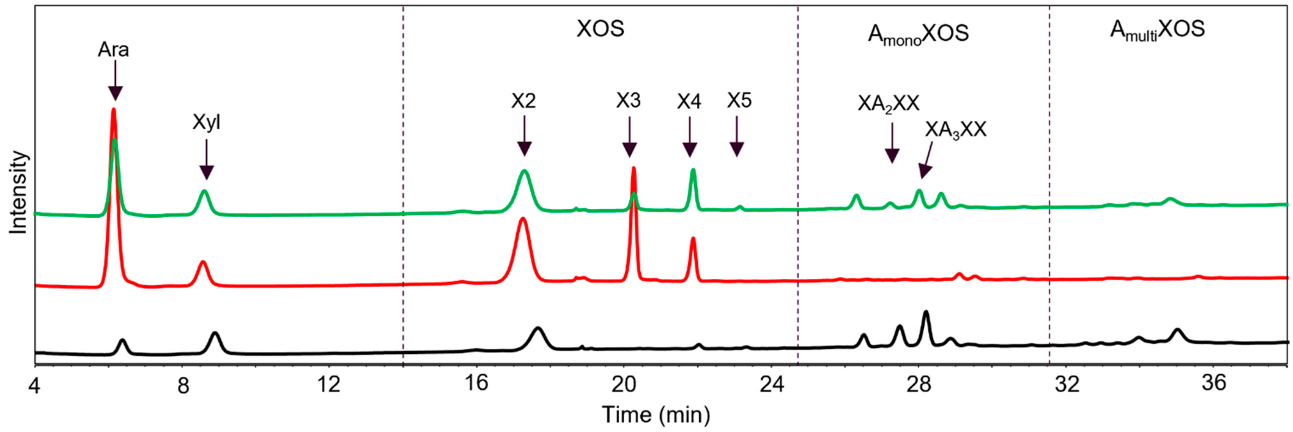

Figure S5. HPAEC reaction products from incubations of GH11 digest of arabinoxylan that was pretreated with ABF-d3, incubated without enzyme (substrate blank) (black), with *Mg*ABF51 (red) and with *Hi*ABF43_36a (green). Incubations were performed with 50 μg mL^{-1} enzyme. Arabinose (Ara), xylose (Xyl), XOS (DP 2-5), monosubstituted AXOS ($A_{mono}XOS$) and multisubstituted AXOS ($A_{multi}XOS$) were detected. Annotated peaks were identified using standards. Experimental conditions are displayed in section 4.2.8.

SI 7. Comparing the rate of HiABF43_36a on $A_{2,3}XX$ and AX.

Data was extrapolated to compare the rate of *Hi*ABF43_36a on $A_{2,3}XX$ to AX. *Hi*ABF43_36a showed maximum activity with at least 0,04 μg mL^{-1} enzyme on $A_{2,3}XX$ (100 μg mL^{-1}, 678,6 g/mol) after 24 h incubation (Figure 9, Section 2.6.2). *Hi*ABF43_36a showed maximum activity with at least 6,25 μg mL^{-1} enzyme on AX by releasing 84 μg mL^{-1} arabinose after 1 h incubation (Figure 8, Section 2.5). Using the equations stated below, ~69 % faster reaction rate on $A_{2,3}XX$ compared to AX was calculated for *Hi*ABF43_36a.

The activity of *Hi*ABF43_36a on $A_{2,3}XX$ was calculated by determining the number of arabinosyl substituents hydrolyzed at minimum enzyme concentration tested that resulted in 100 % conversion per hour incubation as a function of enzyme concentration (Equation S1).

Mol dXyls released from $A_{2,3}XX$ per hour per mL= (Added substrate (g/mL))/(Molecular weight $A_{2,3}XX$(g/mol)) / (Minimum enzyme concentration (μg/mL) * Incubation time (h)) (S1)

The activity of *Hi*ABF43_36a on AX was calculated by mol dXyls cleaved (which is equivalent to arabinose amount released) at minimum enzyme concentration that reached maximum activity per hour incubation as a function of enzyme concentration (Equation S2).

Mol dXyls released from AX per hour= ((Ara released by HiABF43_36a (g/mL))/(Molecular weight Ara(g/mol))) / (Minimum enzyme concentration (μg/mL)* Incubation time (h)) (S2)

Rate on $A_{2,3}XX$ compared to AX was then calculated by dividing their respective amounts of dXyls cleaved per hour (Equation S3).

Rate on $A_{2,3}XX$ compared to AX (%) = Mol dXyls released from AX per hour / mol dXyls released from $A_{2,3}XX$ per hour * 100 % (S3)

SI 8. Synergy between TpABF43_36b and HiABF43_36a and MgABF51 (HPAEC-PAD)

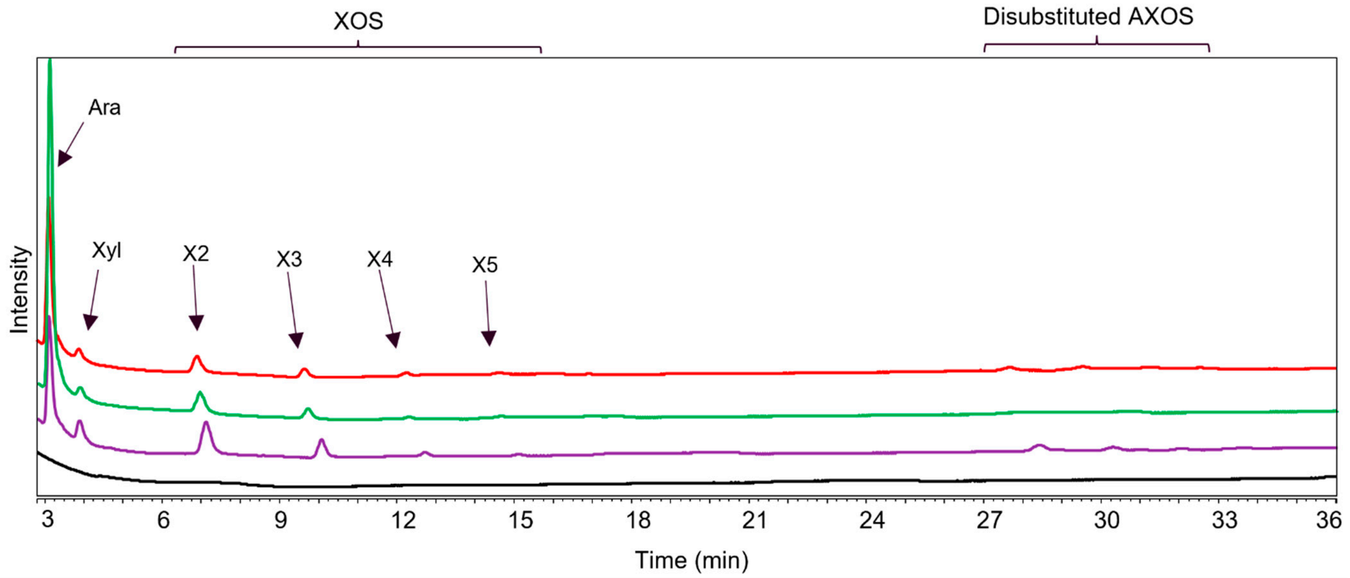

Figure S6. HPAEC reaction product profiles of AX without enzyme (substrate blank) (black), with *Tp*ABF43_36b (purple), with *Tp*ABF43_36b incubated together with *Mg*ABF51 (red) and with *Tp*ABF43_36b incubated together with *Hi*ABF43_36a (green). Reactions were incubated for 23 h and contained 50 µg mL^{-1} enzyme and 1 mg mL^{-1} AX. Arabinose (Ara), xylose (Xyl), XOS (DP 2-5) and disubstituted AXOS were detected.
